# Fission yeast Cdc14-like phosphatase Flp1/Clp1 modulates the transcriptional response to oxidative stress

**DOI:** 10.1038/s41598-023-41869-w

**Published:** 2023-09-06

**Authors:** Juan A. Canete, Sonia Andrés, Sofía Muñoz, Javier Zamarreño, Sergio Rodríguez, Helena Díaz-Cuervo, Avelino Bueno, María P. Sacristán

**Affiliations:** 1 Instituto de Biología Molecular y Celular del Cáncer (IBMCC), Universidad de Salamanca-CSIC, Campus Miguel de Unamuno, 37007 Salamanca, Spain; 2 Departamento de Microbiología y Genética, Universidad de Salamanca, Campus Miguel de Unamuno, 37007 Salamanca, Spain; 3 Present Address: Axentiva Solutions SL, 08036 Barcelona, Spain

**Keywords:** Cell biology, Genetics, Molecular biology

## Abstract

Reactive oxygen species (ROS) are an important source of cellular damage. When ROS intracellular levels increase, oxidative stress takes place affecting DNA stability and metabolic functions. To prevent these effects, stress-activated protein kinases (SAPKs) delay cell cycle progression and induce a transcriptional response that activates antioxidant mechanisms ensuring cell adaptation and survival. Fission yeast Cdc14-like phosphatase Flp1 (also known as Clp1) has a well-established role in cell cycle regulation. Moreover, Flp1 contributes to checkpoint activation during replication stress. Here, we show that Flp1 has a role in fine-tuning the cellular oxidative stress response. Phosphorylation-dependent nucleolar release of Flp1 in response to oxidative stress conditions plays a role in the cellular transcriptional response. Thus, Flp1 ablation increases the transcriptional response to oxidative stress, in both intensity and duration, upregulating both Atf1/Pcr1- and Pap1-dependent stress induced genes. Remarkably, we found that Flp1 interacts with the Atf1/Pcr1 complex with Pcr1 acting as a direct substrate. Our results provide evidence that Flp1 modulates the oxidative stress response by limiting the Atf1/Pcr1-mediated transcription.

## Introduction

All aerobic organisms deal with reactive oxygen species (ROS)-mediated cell damage. ROS are natural byproducts of oxygen metabolism produced during respiration in aerobic organisms and are also triggered by external environmental factors. In eukaryotes, ROS induce the activation of stress-activated protein kinases (SAPKs), a subfamily of mitogen-activated protein kinases (MAPKs), that guarantee adaptation to stress and cell survival^1^. In *Schizosaccharomyces pombe*, a unique SAPK pathway responds to different forms of stress, including osmotic, thermal and oxidative stresses. In this pathway, a central MAPK called Sty1/Spc1 (hereafter referred to as Sty1) is activated under stress conditions. Sty1 is able to induce the activation of several transcription factors that start the transcription programs of both global response genes, those that are expressed under general stress conditions, and specific response genes, those expressed only under certain stress conditions^2–5^. Sty1 is activated by the phosphorylation of Thr-171 and Tyr-173 residues, which is carried out by MAPKK Wis1^3, 6, 7^. In turn, Wis1 is activated by two MAPKKKs, Wis4 and Win1^8–10^. Among the effectors of Sty1 is Atf1, a basic Zipper (bZIP)-containing transcription factor, which is responsible for transcriptional responses to stress conditions. Direct phosphorylation of Atf1 by Sty1 is necessary to activate transcription^11, 12^. Atf1 is homologous to ATF-2, a substrate for the human SAPKs p38 and C-Jun N-terminal kinases, proving the conservation of the stress response kinases throughout evolution^4, 13, 14^. Atf1 heterodimerizes with Pcr1, another bZIP transcription factor, and the resulting Atf1/Pcr1 complex is primarily involved in the induction of Sty1-dependent genes in oxidative stress conditions^11, 15, 16^.

The amplitude and duration of SAPK-mediated transcriptional signaling are tightly regulated in the oxidative stress response^17^. The inactivation of Sty1 is triggered thanks to the contribution of two tyrosine phosphatases, Pyp1 and Pyp2, and two serine/threonine phosphatases, Ptc1 and Ptc3, that remove phosphates from the Tyr-173 and Thr-171 residues of Sty1, respectively^2, 3, 7, 18, 19^. The attenuation of Sty1 signaling is mediated through the Sty1-Atf1 pathway, which constitutes a negative feedback system. Moreover, it has been shown that an additional phosphatase, Ptc4, deactivates the mitochondrial pool of activated Sty1^20^.

In adittion to Sty1 MAPK, the cellular response to oxidative stress also entails the action of Pap1, another bZIP transcription factor, homologue to mammalian c-Jun, that in response to oxidative stress accumulates into the nucleus to trigger a specific antioxidant gene response^21^. Both Sty1-Atf1 and Pap1 pathways regulate the expression of both distinct and overlapping sets of genes in response to different levels of oxidative stress^11, 22, 23^. Moreover, Sty1-Atf1 could negatively regulate Pap1 target genes^22^.

Flp1/Clp1 phosphatase (hereafter referred to as Flp1), the ortholog of *S. cerevisiae* Cdc14, is a non-essential protein important for the proper regulation of mitotic exit and cytokinesis in *S. pombe,* by reversing specific Cdk1 substrate phosphorylation during anaphase^24–27^. In normal cell cycle conditions, Flp1 is sequestered within the nucleolus and the spindle pole body (SPB) during interphase. Then, before anaphase, it is released from sequestration to access its substrates and carry out its functions^24, 25^. Likewise, it has been shown that Flp1 undergoes dynamic subcellular localization changes in response to specific stress conditions. Thus, under replicative stress, Flp1 is released from the nucleolus into the nucleus to regulate the full activation of the checkpoint kinase Cds1^28^. It has been also observed that interphase Flp1 is relocalized from the nucleolus to the nucleoplasm in response to oxidative stress, but not to osmotic or thermal stress^29^. The redistribution of Flp1 under these two genotoxic stress conditions is controlled by specific and complex phosphoregulatory networks, in which different kinases such as Cds1, Chk1, Pmk1 and Cdk1 seem to be involved^28, 29^. Although the role of nucleoplasmic Flp1 accumulation upon replicative stress conditions is already known^28^, the function of nuclear Flp1 under oxidative stress is still to be discovered. In this work, we have explored the functional meaning of Flp1 subcellular relocalization from the nucleolus to the nucleus under oxidative conditions caused by H_2_O_2_ treatment and found that in cells lacking Flp1 phosphatase, the SAPK-mediated transcriptional induction of specific oxidative stress response genes is upregulated in both magnitude and duration. Significantly, Flp1 interacts in vivo with the Atf1/Pcr1 transcription complex and dephosphorylates Pcr1 in vitro. Our findings suggest that the cell cycle regulator Flp1 phosphatase contributes to the fine-tuning of the oxidative stress response in fission yeast by acting on the Atf1/Pcr1 transcriptional complex.

## Results

### Loss of flp1 phosphatase partially rescues the sensitivity of SAPK mutants to oxidative stress and upregulates the stress-related transcriptional response

First, it was confirmed that oxidative stress, caused by 1 mM H_2_O_2_ treatment, induced the release of Flp1-GFP from the nucleolus to the nucleoplasm and that this change of location depended on Flp1 phosphorylation^29^ (Supplementary information Fig. S1a,b). A complex phosphoregulation of Flp1 governs this relocalization. It has been reported that Cds1 and/or Chk1 kinases preferentially act upon replicative stress conditions, whereas Pmk1 and Cdk1 phosphorylate Flp1 upon H_2_O_2_ treatment^28, 29^. Importantly, however, mutation of all nine Cds1/Chk1-RxxS phosphosites in Flp1 (Flp1-9A-GFP mutant, Supplementary information Fig. S1c) was enough to prevent the nucleolar release of the Cdc14-like phosphatase in response to oxidative stress in 95% of cells (Supplementary information Fig. S1), as previously observed upon induction of replicative stress^28^. This percentage of cells showing nucleolar Flp1 retention was higher than that observed in Flp1-6A-GFP, in which only six Flp1 RxxS phosphosites were mutated (Supplementary information Fig. S1). These data indicate that phosphorylation of Flp1 at RxxS phosphosites regulates its relocalization from the nucleolus to nucleoplasm also under oxidative stress conditions.

**Figure 1:**
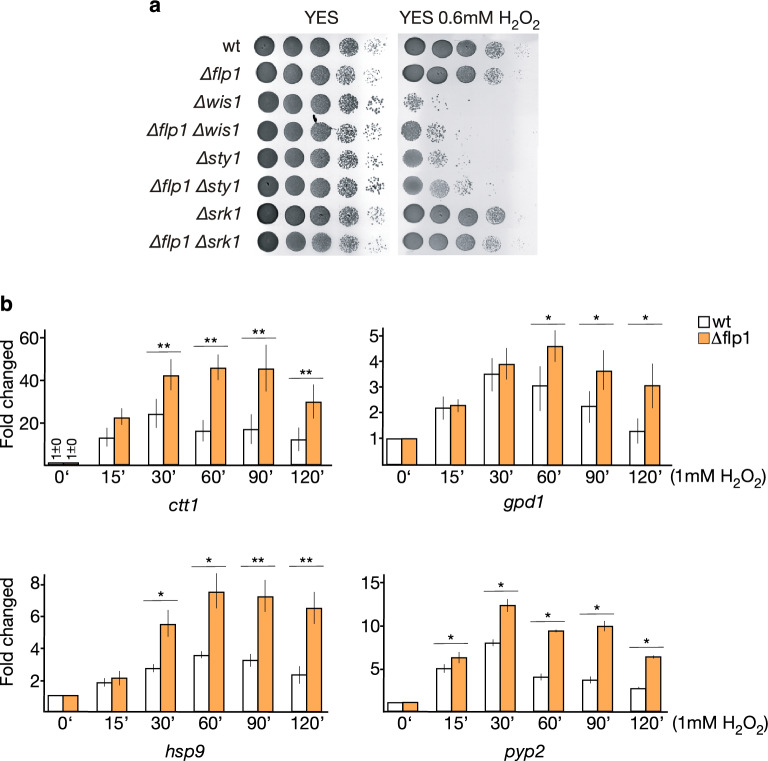
Loss of Flp1 phosphatase alters the cellular response to oxidative stress. (**a**) *flp1* deletion partially rescues sensitivity of *Δwis1* and *Δsty1* strains to chronic presence of H_2_O_2_. Tenfold serial dilution of indicated strains were spotted onto YES-agar plates without or with 0.6 mM H_2_O_2_ and incubated at 30 °C. (**b**) Altered transcriptional profiles in Flp1-deficient cells in response to oxidative stress. Total RNA of samples taken at indicated time points from wild-type and  Δ*flp1* cells treated with 1 mM H_2_O_2_ was extracted and reverse transcribed using a mix of oligo(dT) and random hexamers primers. mRNA levels of *ctt1*^+^, *gpd1*^+^, *hsp9*^+^ and *pyp2*^+^ genes were measured by qPCR and normalized with ribosomal 18S mRNA. In both wild-type and Flp1-deficient cells, the amount of each mRNAs at time 0’ (untreated conditions) was considered 1 and the rest of values were determined with respect to this value. Data were expressed as means SD in triplicate. Asterisks indicate statistical significance (*P < 0.05; **P < 0.005, *t*-test unpaired) versus wild- type.

In response to oxidative stress, Sty1 is activated by Wis1 kinase to induce the activation of several transcription factors that will induce transcription programs of both global and specific oxidative stress response genes^18, 19, 23, 30^. Moreover, Sty1 activates Srk1 kinase to prevent the transition from G2 to mitosis by direct inhibitory phosphorylation of Cdc25, a key mitotic activator^31, 32^*.* To gain insight into the biological function of the nuclear Flp1 accumulation under oxidative stress conditions, we first performed a genetic interaction analysis on stress activated Wis1, Sty1, and Srk1 kinases. Deletion of either *wis1* or *sty1*, but not *srk1,* genes decreased tolerance to oxidative stress (Fig. 1a). Interestingly, the abrogation of *flp1* did not cause additional effects but rather a weak rescue of the sensitivity of *wis1* and *sty1* mutants to the chronic presence of H_2_O_2_ (Fig. 1a). Moreover, Pap1 absence completely suppressed the growth recovery of *∆flp1 ∆sty1* cells, while the lack of Pcr1 and Atf1 increased it (Supplementary information Fig. S2). These results confirm the crosstalk between the Wis1-Sty1-Atf1 and Pap1 signaling routes and suggest Flp1 may have a role in the negative modulation of the oxidative stress response likely through the Sty1-Atf1 pathway.

Many genes are involved in oxidative stress response, whose expression is directly stimulated by the Sty1-Atf1 pathway^30^. Atf1 is a Zip-family transcription factor, activated by Sty1-mediated phosphorylation, essential to transcription initiation during stress^11, 12^. The ability of Flp1 to partially rescue stress response defects in ∆*sty1* and ∆*wis1* mutants prompted us to test whether it could influence the induction of Sty1-Atf1-mediated oxidative stress genes, such as *ctt1*^+^, *gpd1*^+^, *hsp9*^+^, and *pyp2*^+^, coding for catalase, glycerol-3P-dehydrogenase, heat shock protein 9, and a tyrosin phosphatase, respectively. Consequently, we performed a comparative quantitative real-time PCR analysis (qPCR) to check the expression of these genes in both wild-type and *∆flp1* cells under oxidative stress conditions caused by a 1 mM H_2_O_2_ treatment. As shown in Fig. 1b, and confirming previous observations^30^, mRNA expression levels of all these genes increased in wild-type cells at early incubation times (15–30 min) and progressively recovered thereafter until pre-stress levels were reached. However, in cells lacking the *flp1*^+^ gene, the mRNA levels were not only higher after a 15-to-30-min treatment but were also sustained for a longer duration as compared to wild-type cells. In fact, in the absence of Flp1, the cells did not recover initial mRNA expression levels (0 min time point) during the entire treatment. Since the transcription levels of these genes did not increased in cells lacking *flp1* under normal conditions, compared with wild-type cells (Supplementary information Fig. S3a), we concluded that these differences between wild-type and *∆flp1* cells were specific to an oxidative stress response.

**Figure 2:**
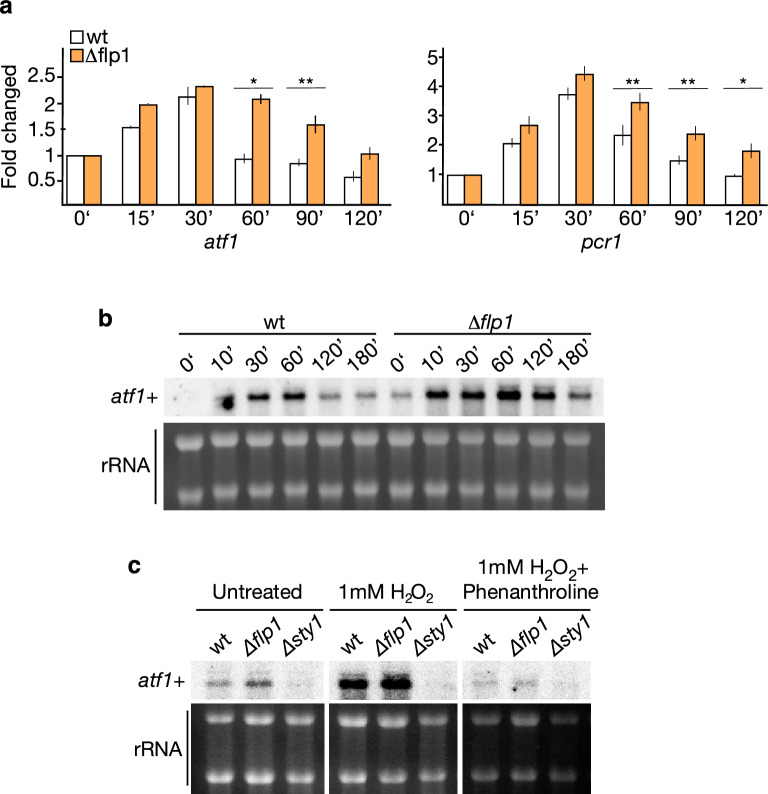
Depletion of Flp1 alters the transcriptional profile of *atf1*^+^ and *pcr1*^+^ transcription factors in response to oxidative stress. (**a**) mRNA levels of *atf1*^+^ and *prc1*^+^ were measured by qPCR from total RNA extracted from wild-type and *Δflp1* strains growing in YES medium containing 1 mM H_2_O_2_. Total RNAs were prepared from treated cells at the time points indicated. mRNA levels were normalized with ribosomal 18S mRNA and determined with respect to time 0’ value, which was considered as 1. Data were expressed as means SD in triplicate. Asterisks indicated statistical significance (*P < 0.05; **P < 0.005, *t*-test unpaired) versus wild-type. (**b**) mRNA levels of *atf1*^+^ were measured by northern blot from total RNA extracted from wild-type and Δ*flp1*strains growing in YES medium containing 1 mM H_2_O_2_. Total RNAs were prepared at the time points indicated and processed for Northern blot analysis. Loading control are rRNAs stained with methylene blue. Full-length blots are shown in Supplementary Fig. S8. (**c**) Exponentially growing cultures of strains indicated were treated with either 1 mM H_2_O_2_ or 1 mM H_2_O_2_ /1,10-phenanthroline (a potent transcription inhibitor). Total RNA was obtained, separated by electrophoresis and northern blots were assayed with a specific *atf1*^+^ probe. Expression of *atf1*^+^ transcription factor gene in ∆*flp1* is abolished when treated with 1,10-phenanthroline showing that it is regulated only at the transcriptional level. Loading control are rRNAs stained with methylene blue. Full-length blots are shown in Supplementary Fig. S8.

It has been shown that under H_2_O_2_ treatment, Atf1 regulates the transcription of Sty1-dependent genes by forming a heterodimer with Pcr1, another basic-leucine ZIP transcription factor^15, 16, 33^. Both *Atf1* and *Pcr1* transcripts are also induced in response to H_2_O_2_ treatment^23, 30, 34^. Thus, we also analyzed the induction of these two transcripts upon oxidative stress conditions in both wild-type and cells lacking *flp1*. We found that the absence of *flp1* did not significantly affect the expected transcriptional upregulation of these two genes at early incubation times (15–30 min) but did affect their recovery kinetics, which were much more attenuated compared to wild-type cells (Fig. 2a), as previously observed for genes *ctt1*^+^, *gpd1*^+^, *hsp9*^+^ and *pyp2*^+^ (Fig. 1b). In the case of *atf1*^+^, these results were confirmed by Northern blot analysis (Fig. 2b,c). Expression analyses performed in the presence of phenanthroline, a potent transcriptional inhibitor^35^, confirmed that the increase in *atf1* mRNA levels was not due to an increase in basal mRNA stability but to an increase in gene expression (Fig. 2c). Moreover, Flp1-mediated modulation of the transcriptional response to oxidative stress depended, as expected, on the activation of the Sty1 MAP kinase, as *atf1*^+^ transcription was comparably repressed in ∆*sty1* and ∆*sty1*∆*flp1* mutant cells (Supplementary information Fig. S3b). These findings highlight that, in fission yeast, nucleoplasmic Flp1 can negatively control the Sty1-Atf1-dependent transcriptional response to oxidative stress conditions.

**Figure 3:**
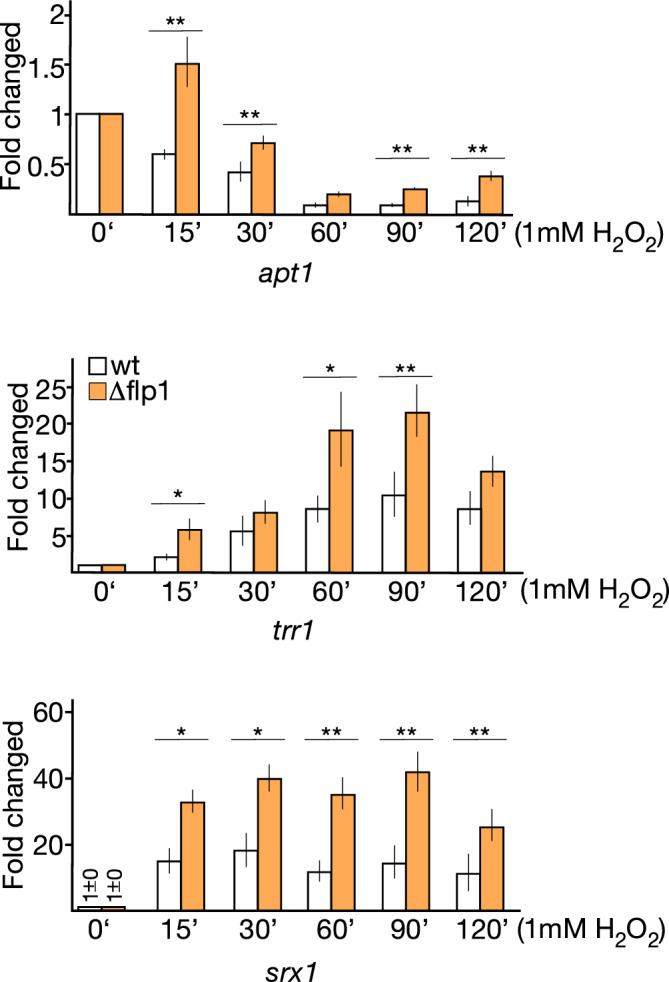
Depletion of Flp1 alters the transcriptional profile of *apt1*^+^, *trr1*^+^ and *srx1*^+^ genes in response to oxidative stress. mRNA levels of *apt1*^+^, *trr1*^+^ and *srx1*^+^ were measured by qPCR from total RNA extracted from wild-type and *Δflp1* strains growing in YES medium containing 1 mM H_2_O_2_. Total RNAs were prepared from treated cells at the indicated time points. mRNA levels were normalized with ribosomal 18S mRNA and determined with respect to time 0´ value, which was considered as 1. Data were expressed as means SD in triplicate. Asterisks indicated statistical significance (*P < 0.05; **P < 0.005, *t*-test unpaired) versus wild-type.

In addition to Atf1 and Pcr1, the bZIP Pap1 transcription factor is likewise connected to the oxidative stress response^11, 21, 22^. To investigate whether Flp1 also regulates the Pap1-dependent expression genes such as *apt1*^+^ and *trr1*^+^ coding for an adenine phosphoribosyltransferase, and a thioredoxin reductase, respectively^21, 36, 37^, comparative qPCR analyses were performed to examine the expression of these genes in both wild-type and *∆flp1* cells under 1 mM H_2_O_2_ treatment. In the case of *apt1*^+^, we observed that the reported mRNA level reduction in response to oxidative stress^30^ was significantly reduced in ∆*flp1* mutant cells, and even an increase of basal levels was found at the earliest incubation time (Fig. 3). For *trr1*^+^, whose levels increase in response to H_2_O_2_ treatment, the absence of *flp1* significantly acentuated its transcriptional upregulation compared to wild-type cells (Fig. 3). The same was observed when examining *srx1*^+^ mRNA encoding a sulfiredoxin reductase, which expression depends on both Atf1 and Pap1 transcription factors^38^. These results strongly suggest that the lack of Flp1 phosphatase also affects the Pap1-dependent pathway in response to oxidative stress conditions. Since Sty1-Atf1 and Pap1 come into two cross-talking oxidative stress activated pathways, between which Atf1 could negatively regulate Pap1 activity^22^, the role of Flp1 on the regulation of Pap1-dependent genes could be indirect, mediated by the regulation of Atf1/Pcr1.

Taken together, the above results suggest that under oxidative stress conditions, Flp1 phosphatase is released from the nucleolus to modulate the transcriptional response of the cell. If this was the case, the *flp1-9A-GFP* mutant, unable to exit the nucleolus in response to 1 mM H_2_O_2_ treatment, should have a transcriptional response similar to ∆*flp1* mutant. Therefore, we next performed qPCR assays to check the expression of several oxidative stress-related genes in both wild-type and *flp1-9A-GFP* mutant cells under 1 mM H_2_O_2_ treatment. As shown in Supplementary information Fig. S4, cells expressing *flp1-9A-GFP* mutant recapitulate the transcriptional profile of ∆*flp1* mutant cells for genes such as *atf1*^+^, *pcr1*^+^, *ctt1*^+^, *gpd1*^+^, and *srx1*^+^.

### The increase in the transcriptional response to oxidative stress of *∆flp1* mutant cells correlates with a greater survival

**Figure 4:**
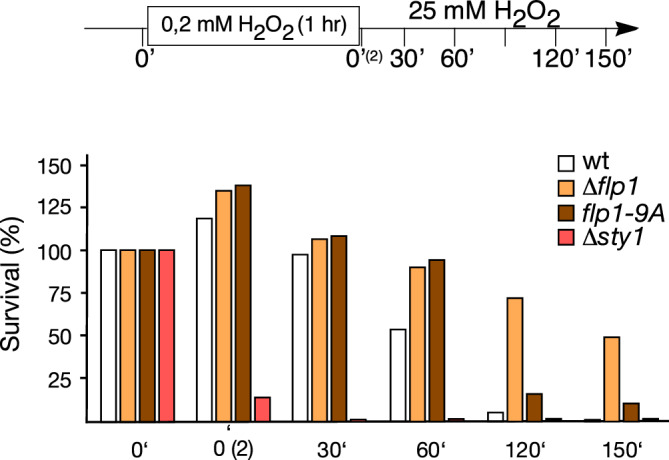
Δ*flp1* cells display greater resistance to acute oxidative stress conditions after exposure to an adaptation period. Asynchronous cultures of indicated strains growing at 30 °C in YES were incubated with 0.2 mM of H_2_O_2_ during 1 h. Then, cells were stressed with 25 mM H_2_O_2_. Cell viability was measured at the indicated time points by plating appropriate dilution of cells onto YES agar plates. The number of viable cells was measured after 3 days incubation at 30 °C. Viability is expressed as a percentage of the number of colonies obtained before the addition of the first dose of H_2_O_2_. Experiment was repeated twice (see Supplementary Information Fig. S5b), and a representative experiment is shown.

Deletion of *flp1*^+^ seems to have no affect in cell viability when cells grow in solid medium containing 0,6 or 1 mM H_2_O_2_ (Fig. 1a and^29^). However, the higher transcriptional induction of all the stress-related genes mentioned above, which combat the harmful effects of oxidative conditions, leads to hypothesize that ∆*flp1* mutant cells should be more resistant upon oxidative stress. To delve further into the biological significance of the regulatory role of Flp1 in oxidative stress, we performed survival assays with cells that were first exposed to a low, non-lethal, dose of H_2_O_2_ to induce basal adaptive response, followed by the incubation with a high dose of the oxidant, that severely compromises growth. After pretreatment with 0.2 mM H_2_O_2_ for 1 h, only 53% of wild type cells were able to survive a subsequent treatment with 25 mM H_2_O_2_ for 1 h, while the percentage of survival of ∆*flp1* and *flp1-9A-GFP* cells was 89% and 93% respectively (Fig. 4). This greater survival was even more prominent after 2 h of 25 mM H_2_O_2_ treatment, time point at which 71% of ∆*flp1* cells survived in contrast to only 5% of the wild type. *flp1-9A-GFP* cells also showed greater survival (16%) than wild type cells although much lower than ∆*flp1* ones (Fig. 4). The increased ability to adapt and survive of ∆*flp1* cells was also observed when after pretreatment with 0.2 mM H_2_O_2_ for 1 h, cells were exposed to an intermediate dose of H_2_O_2_ (2 mM) (Supplementary information Fig. S5a). These data indicate that Flp1 phosphatase is a negative modulator of the adaptive response to H_2_O_2_, which result in a transient resistance to subsequent higher oxidative stress levels.

### Flp1 phosphatase controls the levels and phosphorylation state of Atf1 and Pcr1 trancription factors upon induction of oxidative stress

**Figure 5:**
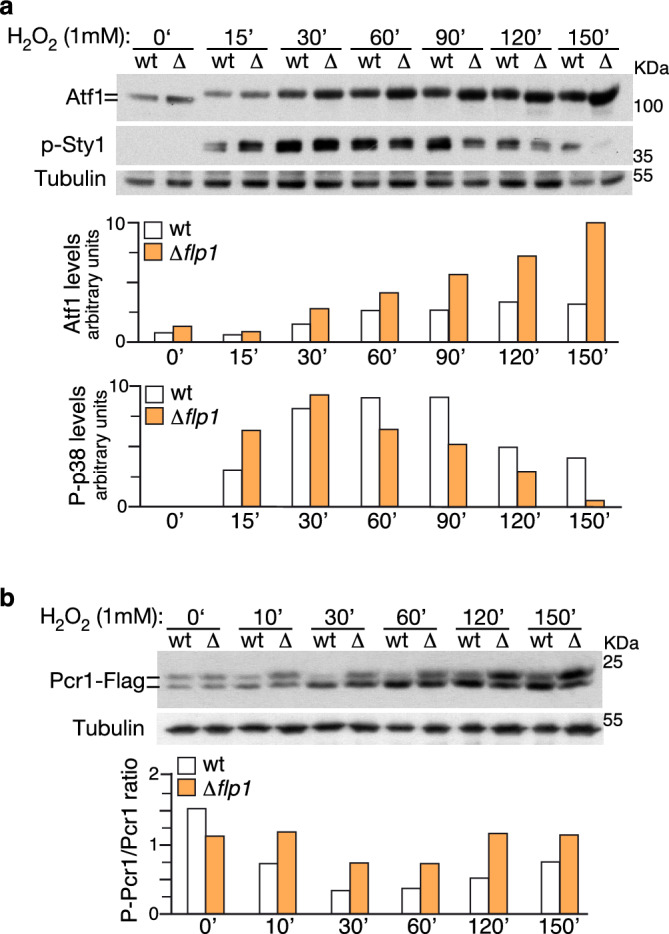
Protein levels and phosphorylation states of Atf1 and Pcr1 transcription factors upon oxidative stress induction. (**a**,**b**) Wild-type and ∆*flp1*cells (∆) expressing a Pcr1-Flag fusion protein were grown in YES medium to mid-log phase and treated with 1 mM H_2_O_2_ for the indicated times. Total extracts were resolved by SDS-PAGE and analyzed by Western blot. (**a**) Atf1 was detected by incubation with a monoclonal anti-Atf1 antibody. Activated Sty1 was detected with anti-phospho-p38 antibody. Tubulin was used as loading control. Normalization of quantified Atf1 and activated Sty1 are shown in bar diagrams. Results from a representative experiment are shown. Blot membranes were cut prior antibodies incubation (Supplementary information Fig. S10). (**b**) Pcr1 was detected by incubation with anti-Flag antibody. Tubulin was used as loading control. Phospho-Pcr1/dephospho-Pcr1 ratios are shown in bar diagrams. Results from a representative experiment are shown. Full-length blots are shown in Supplementary Fig. S10.

Based on the above results, and given that Atf1 and Pcr1 are phosphoproteins, phosphorylated and dephosphorylated respectively in response to oxidative stress, we then analyzed the dynamics of both the protein levels and the state of phosphorylation of the Atf1/Pcr1 complex upon H_2_O_2_ treatment in cells lacking *flp1*^+^. Both wild-type and ∆*flp1* cells expressing Pcr1-Flag were treated with 1 mM H_2_O_2_ and protein extracts were obtained throughout time course experiments. Immunoblot analysis using a monoclonal antibody against Atf1 showed that Atf1 levels gradually increased after 30 min H_2_O_2_ treatment in wild-type cells and that in the absence of *flp1*^+^ this increase was even higher (Fig. 5a). Given that the levels of *atf1* mRNA were upregulated in ∆*flp1* cells when compared to wild-type cells, we deduced that these higher Atf1 levels were largely due to the transcriptional upregulation of the *atf1*^+^ gene expression.

Moreover, as shown by immunoblot analysis, a characteristic shift in Atf1 electrophoretic mobility was observed in wild-type cells early after treatment (15 min time point), due to phosphorylation by Sty1^39^. This shift continued until the 90-min time point, when Atf1 signal began to recover the mobility of its unphosphorylated state (Fig. 5a). In the case of *∆flp1* cells, we observed that the phosphorylation state of Atf1 was similar to that of wild-type cells at early time points (15–30 min). However, unphosphorylated forms were detected earlier (60 min vs 90 min), which is likely to indicate a reduction in the phosphorylation of Atf1 compared to wild-type cells at these time points (Fig. 5a). These data suggest that Sty1-mediated Atf1 phosphorylation is long-term downregulated in the absence of *flp1*^+^. In fact, when testing Sty1 activity in these cells, we unexpectedly found that the dynamics of Sty1 activation during the H_2_O_2_ treatment decreased earlier in the *∆flp1* mutant compared to wild-type cells (Fig. 5a). Since *pyp2*^+^ mRNA, a negative regulatory member of the SAPK pathway involved in the inhibitory dephosphorylation of Sty1, is also increased in *∆flp1* cells (Fig. 1b), we reasoned that it might be responsible for the faster down-regulation of Sty1. We then analyzed Pyp2 protein levels in both wild-type and *∆flp1* cells by immunobloting. Indeed, we observed moderately higher levels of Pyp2 in *∆flp1* cells than in the wild-type ones, especially at the last time points (Supplementary information Fig. S6). From these data, we conclude that although the overall magnitude and dynamics of the Sty1-dependent transcriptional response is increased in cells lacking the *flp1*^+^ gene compared to wild-type cells, the long-term result is a faster recovery to pre-stress Sty1 activity levels.

It has been shown that Pcr1, the partner of Atf1 regulating the expression of most of the Sty1-dependent genes under oxidative stress conditions^15, 16^ is dephosphorylated upon H_2_O_2_ treatment^16^. Thus, upon oxidative stress, hyperphosphorylation of Atf1 parallels dephosphorylation of Pcr1, both dependent on Sty1 kinase activity^16^. Immunoblot assays allowed us to corroborate previous findings regarding the accumulation of Pcr1 protein upon H_2_O_2_ -mediated oxidative stress^40^, which was higher in *∆flp1* cells, correlating with the higher levels of *pcr1* mRNA in ∆*flp1* cells compared to wild-type ones under these stress conditions (Fig. 2a). Interestingly, in cells lacking Flp1 phosphatase, Pcr1 never reached the dephosphorylation level observed in wild-type cells, even its phosphorylated forms were the most predominant at the later time points analyzed during the H_2_O_2_ treatment (Fig. 5b). These data suggest that Flp1 could be, at least in part, the phosphatase responsible for Pcr1 dephosphorylation under oxidative stress conditions.

**Figure 6:**
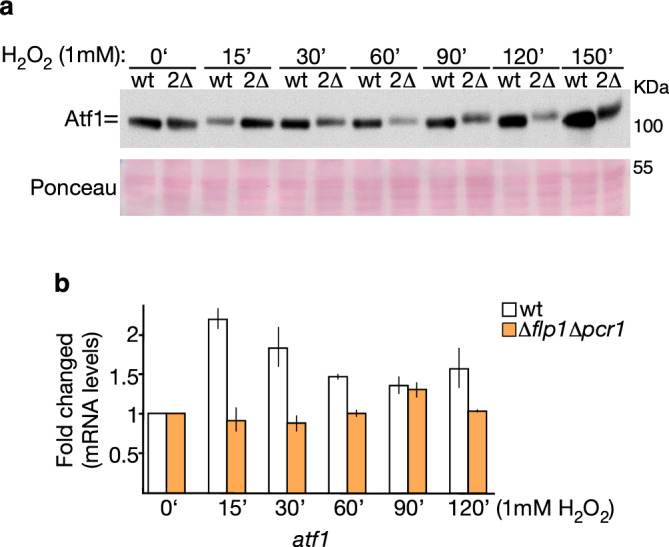
Protein and mRNA levels of Atf1 transcription factor in cells lacking both *flp1* and *pcr1* genes upon oxidative stress induction. (**a**,**b**) Wild-type and ∆*flp1* ∆*pcr1*mutant cells (2∆) were grown in YES medium to mid-log phase and treated with 1 mM H_2_O_2_ for the indicated time points. (**a**) Total protein extracts were resolved by SDS-PAGE and analyzed by Western blot. Atf1 was detected by incubation with a monoclonal anti-Atf1 antibody. Ponceau staining was used as loading control. Full-length blots are shown in Supplementary Fig. S12. (**b**) Total RNAs were prepared from untreated (0´) and treated cells at the time points indicated. mRNA levels of *atf1*^+^ were measured by qPCR, normalized against ribosomal 18S mRNA and determined with respect to time 0´ values, which were considered as 1. Data represent the average of two biological replicates.

It has been reported that Atf1 stability is modulated by Sty1 phosphorylation and binding with Pcr^40^. Since phosphorylation status of Atf1 in *∆flp1* cells differs from that of wild-type ones at later time points, when it appears less phosphorylated (Fig. 5a), and based on the fact that Pcr1 shows increased phosphorylation in the absence of Flp1, we studied whether the higher Atf1 levels in *∆flp1* cells are due to its interaction with Pcr1. As shown in Fig. 6, the higher increase of both protein and mRNA Atf1 levels, observed in *∆flp1* cells under oxidative stress conditions, was abolished in the double mutant *∆flp1 ∆pcr1*, indicating that Atf1 abnormalities observed in *∆flp1* cells are mediated by Pcr1.

### Flp1 interacts with and dephosphorylates the transcription factor Pcr1

**Figure 7:**
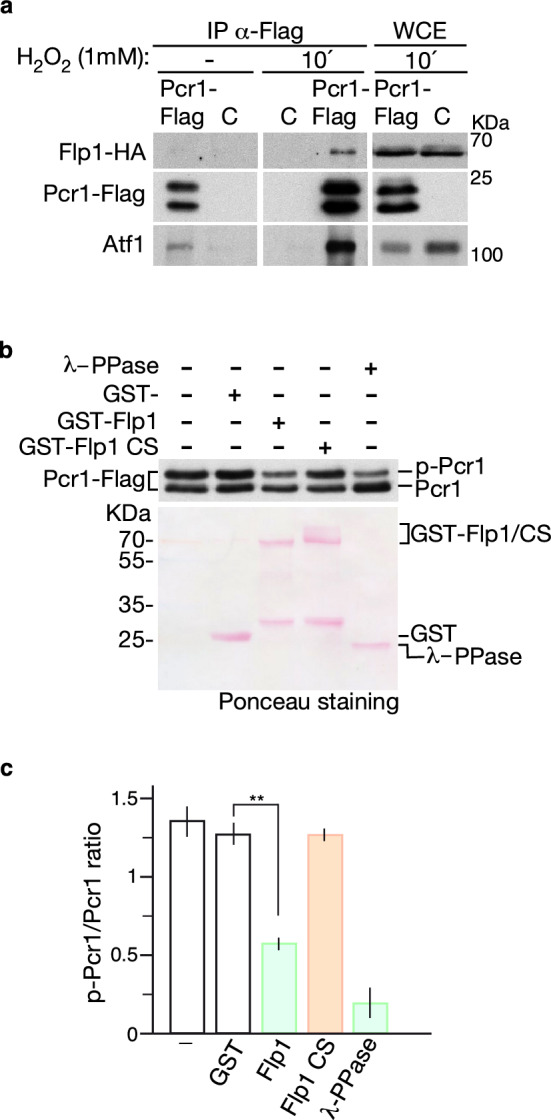
Pospho-Pcr1 protein is a substrate of Flp1 phosphatase. (**a**) Coimmunoprecipitation of Pcr1-Flag and Flp1-HA fusion protein from yeast extracts obtained from indicated strains untreated or treated with 1 mM H_2_O_2_ for 10 min and incubated with formaldehyde for 20 min before processing. Anti Flag antibody was used to immunoprecipitate Pcr1-Flag fusion protein. Immunoprecipitates were analyzed by immunoblot using anti-Flag, anti-HA and anti-Atf1 antibodies. *IP* immunoprecipitation, *WCE* whole cell extracts; C, negative control: untagged strain. Cropped gels are shown. Unprocessed original scans of blots, cut prior antibodies incubation, are shown in Supplementary information Fig. S13. (**b**) Flp1 dephosphorylates Pcr1 in vitro. Pcr1-FLAG was immunoprecipitated from protein extracts prepared from ∆*flp1* cells treated with 1 mM H_2_O_2_ for 10 min. After washing, Pcr1-FLAG immunoprecipitates were incubated with GST-Flp1, the phosphatase dead mutant GST-Flp1-CS, GST alone or λ phosphatase. Samples were resolved by SDS-PAGE and detected by immunoblot with anti-FLAG antibody or ponceau staining. Full-length blots are shown in Supplementary Fig. S13. (**c**) Quantification of in vitro phosphatase assays performed as described in (**b**). Bars (Phospho-Pcr1/dephospho-Pcr1 ratio) indicate the average from four independent experiments. **P < 0.005, as calculated by unpaired Student’s *t* test.

To investigate whether Flp1 directly regulates the phosphorylation state of Pcr1 in response to oxidative stress, we first analyzed whether Flp1 and Pcr1 proteins interact in vivo. We exposed cell cultures to 1 mM H_2_O_2_ and immunoprecipitated Pcr1-Flag tagged protein from a strain also expressing Flp1 protein tagged with the HA epitope. The interaction was also checked in untreated cells. To preserve the characteristic labile interactions between phosphatases and their substrates, as well as Atf1-Pcr1 complexes, we used formaldehyde to cross-link protein complexes in cultured cells. Immunoprecipitated Flag-Pcr1 was resolved by SDS-PAGE gels followed by immunoblotting with the corresponding antibodies. Flp1 was clearly detected in the Flag-Pcr1 immunoprecipitates obtained from cells treated with H_2_O_2_ (Fig. 7a), indicating that Flp1 interacts with Pcr1 under oxidative stress conditions.

Finally, to examine whether Pcr1 is a direct substrate of Flp1, we performed in vitro phosphatase assays using Pcr1-Flag immunoprecipitates obtained from exponentially cells grown in YES medium and recombinant GST-Flp1 or GST-Flp1-CS (a catalytically inactive form) fusion proteins purified from the proper *S. pombe* strains. As shown in Fig. 7b, incubation of Pcr1-Flag immunoprecipitates with λ-PPase, used as a positive control, and wild-type Flp1, but not with the catalytically inactive GST-Flp1-CS form, resulted in some degree of Pcr1 dephosphorylation, as shown by the reduction level of the phosphorylated Pcr1 upper band (Fig. 7c), indicating that Flp1 is able to dephosphorylate Pcr1 in vitro. All these data support a role for Flp1 in the dephosphorylation of Pcr1 during oxidative stress.

## Discussion

Eukaryotic cells are continuously exposed to ROS during normal aerobic metabolism. However, an excess of ROS causes oxidative stress by damaging all cellular components including DNA, proteins, and lipids. Cells have developed response mechanisms to avoid these harmful effects, maintain ROS homeostasis and maximize survival. One of the majors signaling pathways, SAPK pathway, reprograms gene expression to induce proteins with stress protection function. In *S. pombe*, Sty1 MAPK, the p38 kinase ortholog in mammals, is activated to stimulate gene expression via the Atf1 transcription factor which is closely related to ATF2 in humans^18^. Sty1-mediated phosphorylation of Atf1 is essential to promote transcription initiation^11^. Atf1 forms a heterodimer with the transcription factor Pcr1, also a basic zipper (bZIP) protein, which seems to allow proper recognition of Atf1-binding sites at most promoters^11^. Pcr1 is also a phosphoprotein that is specifically dephosphorylated in a Sty1-dependent manner under oxidative stress^16^, although the functional meaning of this posttranscriptional modification is still unknown. Moreover, the pathway driven by the bZIP transcription factor Pap1 is also involved in the cellular response to oxidative stress^21^. Pap1 contributes with Atf1-Pcr1 to properly activate essential antioxidant genes and, in turn to get a full transcriptional response^11, 22, 23, 41^.

The conserved Cdc14 phosphatase family, implicated in the reversal of Cdk and MAPK substrate phosphorylation to regulate cell cycle progression in yeast^42–44^, is also involved in the response to different types of cellular stress. Moreover, it has been described that the *S. pombe* Cdc14 ortholog, Flp1, whose activity is largely controlled by subcellular location, participates in the cellular response to replicative stress^28^. Thus, by exiting its interphase nucleolar localization and its subsequent accumulation in the nucleoplasm, Flp1 contributes to the full activation of the replicative stress checkpoint^28^. One mammalian ortholog, Cdc14B, also responds to DNA damage insults exiting the nucleolus to participate in the DNA damage checkpoint activation^45^. Moreover, it has been shown that Flp1 also responds to oxidative stress conditions by exiting the nucleolus and accumulating in the nucleoplasm^29^, although the biological meaning of this localization change is not yet understood. Nonetheless, we report here that Flp1 phosphatase modulates the transcriptional response to oxidative stress, likely acting on the Atf1/Pcr1 transcriptional complex.

Although the lack of *flp1*^+^ in *S. pombe* does not sensitize cells to oxidative stress caused by the continuous exposure to intermediate doses of H_2_O_2_ treatment (this work and^29^), we found that the sensitivity of stress-activated MAPKs mutants, such as *∆wis1* and *∆sty1*, to oxidative stress caused by H_2_O_2_ treatment, is partially suppressed when combined with *∆flp1*. Moreover, we also found that both ∆*flp1* and *flp1-9A-GFP* mutants cells survive better than wild-type cells when exposed to acute oxidative stress after pretreatment with low, non-lethal, doses of H_2_O_2_ to allow cells to adapt the response.

This suggests that Flp1 phosphatase plays a role in the regulation of the oxidative stress response, likely acting as a negative modulator of the adaptive response to low levels of ROS in the cellular environment.

It has been reported that the active release of Flp1 phosphatase from the nucleolus to the nucleoplasm is an all-or-none response mediated by the phospho-dependent association of Rad24 with the phosphatase, which avoids returning to the nucleolus upon those environmental conditions. In this scenario, Pmk1/Cdk1 kinases preferentially acting upon oxidative stress^29^, and Cds1/Chk1 the ones that phosphorylate Flp1 in response to replicative stress^28, 29^. However, by using a previously characterized Flp1 mutant, in which all nine Cds1/Chk1-mediated RxxS phosphosites were eliminated (Flp1-9A-GFP)^28^, we found that Cds1/Chk1-related kinases seem also to underlie the nuclear accumulation of the phosphatase in response to oxidative stress. Previous accurate studies have shown that abolishing six Flp1 RxxS phosphosites, although significantly hampered Flp1nucleoplasmic accumulation, was not enough to fully prevent Flp1 release from the nucleolus under H_2_O_2_ treatment, and that this required three additional mutations at the putative Pmk1/Cdk1-dependent TP phosphosites present in Flp1 (Flp1-6A3A-GFP mutant). These observations strongly suggested that phosphorylation of Flp1 at both RxxS and TP sites regulates its relocalization from the nucleolus to the nucleoplasm upon oxidative stress^29^. Additionally, this difference in reported data could be due to the alteration of Cdk/Pmk1-mediated Flp1 phosphorylation sites as a consequence of conformational changes of Flp1 resulting from the nine serine mutations in the Flp1-9A-GFP mutant. However, the fact that Flp1-9A-GFP cells behave as wild-type cells in an unperturbed cell cycle^28^ supports the former postulate.

Several Sty1-dependent mechanisms have been suggested to explain the increase of *atf1*^+^ expression levels in response to stress. This high levels of Atf1 are important for proper adaptation to maximize cell survival under these harmful conditions but not for unstressed cells^40, 46–48^, indicating that normal expression levels of *atf1*^+^ have to be recovered even under persistent stress. Similar to *atf1*^+^, the Atf1-mediated increase in the expression of genes with a stress-protective function decay after a certain time until reaching their characteristic levels of unstressed conditions. Under oxidative stress, lack of Flp1 phosphatase results in a significant increase in the transcription levels of Sty1/Atf1-dependent genes when compared with the levels reached in wild-type cells. Moreover, in *∆flp1* cells, this transcriptional response is also deregulated in time. Thus, *∆flp1* cells respond by a significant higher transcriptional induction, in both magnitude and duration, of key genes such as *ctt1*^+^*, gpd1*^+^*, hsp9*^+^ *and pyp2*^+^ compared to wild-type cells. Analysis of *atf1*^+^ and *pcr1*^+^ induction also showed longer transcriptional kinetics over time, in which the mRNA levels prior to stress were not recovered during the time the analysis lasted. The upregulation of *atf1*^+^ and *pcr1*^+^ transcription factors in *∆flp1* cells most likely account for the upregulation of the stress-activated genes presented in this work. Moreover, upregulation of Pap1- dependent genes in response to oxidative estress was also higher in *∆flp1* cells when comparing with wild-type ones.

Upon the induction of oxidative stress, the lack of Flp1 also results in a significant increase in Atf1 and Pcr1 protein levels, most likely reflecting the also higher *atf1*^+^ and *pcr1*^+^ mRNA levels, an anomalous phosphorylation state of both transcription factors, and intriguingly, a faster deactivation of the Sty1 kinase. Sty1-mediated phosphorylation of Atf1 has a key effect on the stress-activated transcriptional response by establishing proper interactions with the basal transcriptional machinery for transcription initiation^11^. In *∆flp1* cells, the oxidative stress-dependent phosphorylation of Atf1 is similar to that observed in wild-type cells, although dephosphorylated forms are detected earlier in time, which may reflect the earlier deactivation of Sty1 in *∆flp1* cells, most likely due to the higher levels of Pyp2 phosphatase reached at the later time points in these cells as compared to wild-type ones. Parallel to Atf1 phosphorylation, Pcr1 is dephosphorylated upon oxidative stress conditions^16^. Remarkably, Pcr1 dephosphorylation is much less evident in cells lacking Flp1. Since Pcr1 is dephosphorylated in a Sty1-dependent manner, the faster deactivation of Sty1 in *∆flp1* cells could also account for the predominance of phosphorylated forms of Pcr1 at later time points. However, the lack of Pcr1 dephosphorylation in *∆flp1* cells at early time points cannot be explained as a consequence of the downregulation of Sty1 activity. An attractive possibility is that Flp1 modulates the response to oxidative stress, interacting with and dephosphorylating stress-regulated transcription factors. Several lines of evidence shown here strongly suggest that this is the case. First, in cells lacking *flp1*^+^, transcription of Sty1-Atf1-mediated oxidative stress genes is deregulated. Second, Flp1 associates in vivo with the Pcr1-Atf1 complex under oxidative stress conditions. Third, Flp1 dephosphorylates the Atf1-cofactor Pcr1 in vitro and the absence of Flp1 correlates with the lack of Pcr1 dephosphorylation in vivo in response to oxidative stress. Lastly, when we checked cell viability in response to H_2_O_2_ treatment in both *∆pcr1* and *∆pcr1 ∆flp1* mutants we did not observe additive effects (Supplementary Fig. S7), suggesting that the two proteins work in the same pathway.

Previous works have already reported a role for Flp1 in the transcriptional regulation of genes important for mitosis and cytokinesis as well as S phase gene expression, most likely through the association and dephosphorylation of specific transcription factors^49, 50^. Moreover, mammalian Cdc14 paralogs have also been involved in transcriptional regulation. Thus, the mouse Cdc14B paralog, in pluripotent cells, regulates the exit from stemness to differentiation through dephosphorylation and consequent degradation of the UTF1 repressor transcription factor^51^. In addition, the human Cdc14B phosphatase participates in the repression of cell cycle transcription by direct RNA polymerase II regulation^52^.

**Figure 8:**
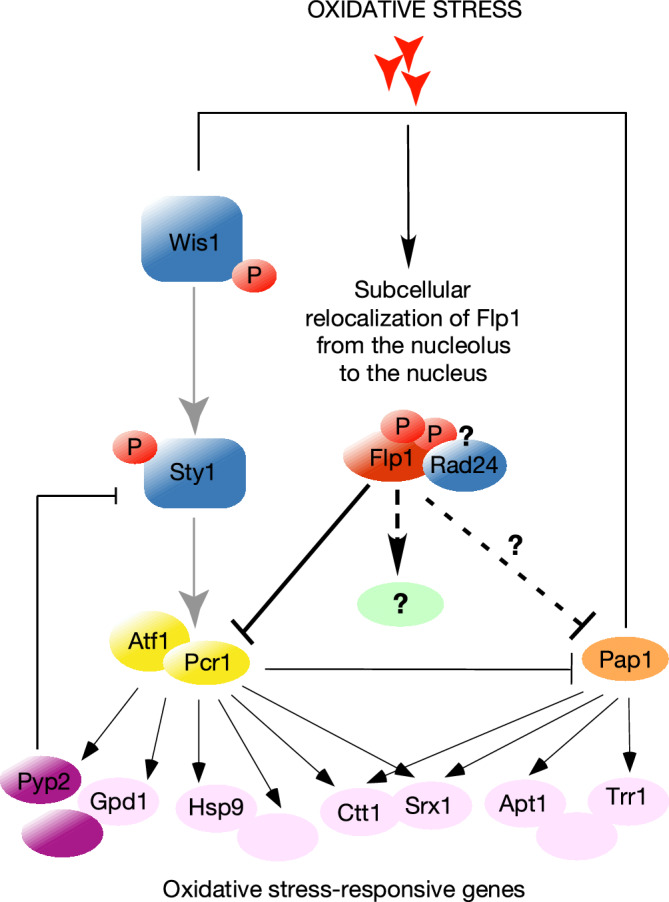
Model of the role of Flp1/Cdc14 in the modulation of the transcriptional response to oxidative stress (See text for details).

Our data support a model in which Flp1 is released from the nucleolus to the nucleoplasm upon oxidative stress through the phosphorylation of its RxxS phosphosites and the consequent association of Flp1 with Rad24. Once released, nuclear Flp1 phosphatase, bound or not to Rad24, interacts with the Atf1/Pcr1 complex, dephosphorylating Pcr1 to modulate the transcriptional response. Flp1 may be involved in the dephosphorylation of Pcr1 in combination with other unknown phosphatases, but it could be also acting on another not yet known target, such as Pap1 itself (Fig. 8). Therefore, we propose that Flp1-phosphatase is a negative regulator of the transcriptional response to oxidative stress acting on its deactivation or maintaining the activity levels of the Wis1/Sty1 pathway within certain limits. How the dephosphorylation of Pcr1 by Flp1 underlies the modulation of the transcriptional response and to understand whether Pap1 is regulated by Flp1 will be subjects of future studies.

## Material and methods

### Fission yeast strains, growth conditions and media

The strains used in this study are listed in Supplementary information Table S1. Media and genetic methods for studying fission yeast were previously described^53^. For plate survival assays, serial dilutions of exponentially growing cultures of strains indicated were plated in media without or with different concentrations of H_2_O_2_ (as indicated in figure legends). Plates were incubated at 30 °C for three days and then scanned.

### Plasmids construction

Plasmids pRepKZ- *flp1*^+^ complete ORFs under the *nmt1* promoter was constructed by PCR amplification from genomic DNA. Transformation was performed by lithium acetate protocol or electroporation and transformants were selected by growing in selective medium.

### RNA analysis

RNA samples for Northern analysis were prepared from 0.5 × 10^9^ cells. Yeast cultures grown at 30 °C to early log phase were keep untreated or exposed to 1 mM H_2_O_2_ for the time specified in the figure legends. Pellets were resuspended in 200 ml RNA extraction buffer (0.1 M EDTA pH 8.0, 0.1 M NaCl, 50 mM Tris pH 8.0) with 200 ml Phenol/Chloroform and 5 ml 10% SDS. Cells were disrupted with glass-beads in a Fast-prep and then spined in a microfuge for 15 min. The aqueous layer was collected and extracted once with Phenol, three times with Phenol/Chloroform and once with Chloroform/Isoamyl alcohol. RNA was precipitated with sodium acetate and isopropanol. RNA pellets were then washed with 70% Ethanol and resuspended in H_2_O. Total RNA (6 μg) was denatured by heating at 70 °C and deionized formamide and formaldehyde was added to each sample. Samples were separated in Formaldehyde 1.2% agarose with ethidium bromide gels and transferred to a Hybond-N membrane (GE Healthcare). 500 nucleotides gene specific probes were obtained by PCR amplification from genomic DNA and labelled with ^32^P-α-dCTP. Transcript levels were quantified according to loading control using Bio-Rad Molecular Personal FX Phosphorimager. For quantitative real-time PCR (qPCR) analysis, total RNAs were purified using the RNeasy minikit (Qiagen), treated with DNase (Invitrogen) and quantified using a NanoDrop spectrophotometer (Isogen). Total RNAs (1 μg) were reversed transcribed into cDNA using iScript reverse transcription supermix (Bio-Rad). qPCRs were performed using the iTaq Universal SYBR green supermix and an iCycler PCR system (Bio-Rad). Relative gene expression was normalized using a stress-independent gene (18S rRNA). Gene specific primers used for qPCRs are indicated in Supplementary information Table S2.

For distinguishing between mRNA synthesis or mRNA stability cells were grown to OD_600_ of 0.4 and then 1,10-phenanthroline dissolved in ethanol was added to cultures (250 μg/ml) to inhibit transcription.

### Protein methods

#### Western blotting analysis

Whole cell extracts were prepared by precipitation with trichloroacetic acid (TCA). *S. pombe* strains were grown in YES medium to OD_600_ of 0.4–0.5 and cells (5 ml) were collected by centrifugation just after the addition of 100% TCA to a final concentration of 10% and washed in 20% TCA. Cell disruption was performed with Glass-Beads in a Fast-Prep and 12.5% TCA. Cell lysates were pelleted by centrifugation at 3.000 rpms and resuspended in 1X LB loading buffer and Tris base. Samples were electrophoretically resolved by SDS-PAGE and transferred to Nitrocellulose membranes using a Bio-Rad transfer unit. Blots were then probed against antibodies indicated. Antibodies used: monoclonal anti-Atf1 (Abcam), anti-HA-HRP conjugated (Miltenyi Biotec), anti-Flag-HRP conjugated (Sigma), anti-Myc-HRP conjugated (Miltenyi Biotec), anti phospho-p38 MAP Kinase (Cell Signaling) and anti-α-tubulin (Sigma-Aldrich).

#### Coimmunoprecipitation

For immunoprecipitation of Flag-tagged Pcr1 protein, *S. pombe* strains expressing Pcr1-Flag and/or Flp1-HA fusion proteins were grown in YES at 30 °C to an OD_600_ of 0.5. Then H_2_O_2_ (1 mM) was added to part of the cultures and after 10 min incubation formaldehyde (1.5% vol/vol) was added for 30 min at room temperature. Cells were pelleted and washed three times with ice cold TBS. Pellets were resuspended in 500 ml lysis buffer (20 mM Tris, pH 8, 100 mM NaCl, 2 mM EDTA and 0.5% NP-40) supplemented with a protease inhibitor cocktail (Complete, EDTA-free, Sigma-Aldrich) and disrupted with Glass-Beads in a Fast-Prep. Cell lysates were clarified by centrifugation at 13.000 rpms and protein concentration was determined using BCA Protein Assay kit (Pierce Chemical, Rockford, IL). Cell extracts (3 mg) were incubated with dynabeads protein G (Invitrogen) bound to monoclonal anti-Flag antibody (Agilent Technologies) for 5 h at 4 °C. Beads were washed four times with lysis buffer and resuspended in loading buffer. Immunoprecipitates were resolved by SDS-PAGE, transferred to Nitrocellulose membranes using a Bio-Rad transfer unit and analyzed with anti-HA-HRP conjugated (Miltenyi Biotec), anti-Flag-HRP conjugated (Sigma) and anti-Atf1 (Abcam) antibodies.

### In vitro phosphatase assays

Pcr1-Flag was immunoprecipitated from cells grown in YES at 30 °C to an OD_600_ of 0.5. Cells were recovered by centrifugation, disrupted with Glass-Beads in the mentioned buffer lysis, and immunoprecipitation was performed as described above. GST-Flp1 and GST-Flp1-CS fusion proteins were purified from *S. pombe* strains containing the corresponding repressible *nmt*1-plasmids. Cells were grown in minimal medium appropriately supplemented (adenine and uracil) and containing thiamine until exponential phase. Induction was performed by washing cells three times with water and inoculated on minimal medium without thiamine at OD_600_ of 0.05. Cultures were allowed to grow at 30 °C for 16 h. GST-Flp1 fusion proteins were purified from these cells as previously described (Díaz-Cuervo 2008). Pcr1-Flag immunoprecipitates were washed in phosphatase buffer (50 mM Imidazol pH 7, 1 mM EDTA, 1 mM DTT) and incubated for 30 min at 30 °C with GST-Flp, GST-Flp1-CS, GST- alone or λ-phosphatase in the above phosphatase buffer. Reactions were stopped by addition of loading buffer and boiling for 5 min at 95 °C. Proteins were resolved by SDS-PAGE, transferred to nitrocellulose membranes and analyzed with anti-Flag-HRP conjugated antibody to detect Pcr1-Flag mobility. Membranes were also analized by ponceau staining to detect GST- fusion proteins.

### Mycroscopy

GFP-, EGFP-, mCherry-, and RFP-tagged strains were grown in YES medium until exponential phase. 1 mM H_2_O_2_ was added during 1 h and the presence of specific fluorescence was detected by fluorescence microscopy using a Thunder Imager (camera, DFC9000; Leyca) microscope.

### Supplementary Information

Supplementary Information.

## Data Availability

The datasets supporting the current study are available from the corresponding authors on request.
