# Supplementary material for: Fission yeast Cdc14-like phosphatase Flp1/Clp1 modulates the transcriptional response to oxidative stress
Source: Sci Rep. 2023 Sep 6;13:14677. doi: 10.1038/s41598-023-41869-w (PMC10482896; doi:10.1038/s41598-023-41869-w)
Supplement: Supplementary file 1 — Supplementary Information. [file 41598_2023_41869_MOESM1_ESM.pdf]

Fission yeast Cdc14-like phosphatase Flp1/Clp1 modulates the transcriptional response to oxidative stress

Juan A. Canete[1,2], Sonia Andrés[1,2], Sofía Muñoz[1,2], Javier Zamarreño[1,2], Sergio Rodríguez[1,2], Helena Díaz-Cuervo[1,2,3], Avelino Bueno[1,2]* and María P. Sacristán[1,2]*

[1]Instituto de Biología Molecular y Celular del Cáncer (IBMCC), Universidad de Salamanca-CSIC, Campus Miguel de Unamuno, 37007 Salamanca, Spain.

[2]Departamento de Microbiología y Genética, Universidad de Salamanca, Campus Miguel de Unamuno, 37007 Salamanca, Spain.

[3]Current affiliation: Axentiva Solutions SL. ES08036 Barcelona, Spain.

*Corresponding authors: msacristan@usal.es and abn@usal.es

Supplementary Information

Supplementary Figure S1. Flp1 phosphatase changes its subcellular localization under oxidative stress in a phosphorylation-dependent manner. (a) Live-cell images of *gar2-mCherry* cells also expressing either Flp1-GFP, Flp1-6A-GFP or Flp1-9A-GFP fusion proteins under unperturbed growth conditions or after treatment with 1mM of H_2O_2 for 1 h. Asterisks indicate Flp1-GFP nucleoplasmic localization. Scale bar, 5 µm. (b) Line scans of Flp1-GFP and Gar2-mCherry fluorescence of the boxed nuclei from de merged images in (a), represented by a white line and spanning 4 µm from the white closed circle. Fluorescence intensity was normalized to the maximum value after background subtraction. (c) Schematic representation of Flp1 showing all nine RxxS putative phosphorylation sites, which are mutated to alanine in the Flp1.9A-GFP mutant. Asterisks indicate the six serine residues that are mutated to alanine in the Flp1.6A-GFP mutant. (d) The graph shows the percentage of nuclei with Flp1-GFP, Flp1.6A-GFP or Flp1.9A-GFP detected in the nucleoplasm both under unperturbed growth conditions and after treatment with 1mM of H_2O_2 for 1h.

Supplementary Figure S2. *flp1*$^{+}$ deletion partially rescues sensitivity of Δ*sty1* strain to chronic presence of H_2O_2 in a *pap1*$^{+}$-dependent manner. Tenfold serial dilution of indicated strains were spotted onto YES-agar plates without or with 0.6 mM H_2O_2 and incubated at 30ºC during 72 h.

Supplementary Figure S3. (a) Expression of stress-responsive genes indicated in unstressed wild-type and Δ*flp1* cells. Total RNA from asynchronous cultures of the indicated strains grown at 30 ºC was extracted and reverse transcribed using a mix of oligo(dT) and random hexamers primers. mRNA levels of *cttl*$^{+}$, *gpd1*$^{+}$, *hsp9*$^{+}$ and *pyp2*$^{+}$ genes were measured by qPCR and normalized with ribosomal 18S mRNA. The amount of mRNAs relative to the wild-type was expressed as means SD in triplicate. Asterisks indicate statistical significance (*, P<0.05; *t*-test unpaired) versus wild-type. (b) Exponentially growing cultures of strains indicated at 30 ºC were treated with 1mM H_2O_2 for 1 hour. Total RNA was extracted, separated by electrophoresis and rRNAs stained with methylene blue were used as loading control. Northern-blot analysis was used to measure the expression levels of *atf1*$^{+}$ in the indicated strains. Full-length blots are shown in Supplementary Fig. S9.

Supplementary Figure S4. *Flp1-9A-GFP* strain, expressing a nonphosphorylatable *flp1* mutant unable to exit the nucleolus under oxidative stress conditions, shows altered the transcriptional profile of genes such as *atf1*$^{+}$, *pcr1*$^{+}$, *ctt1*$^{+}$, *gpd1*$^{+}$ and *srx1*$^{+}$ induced in response to oxidative stress. mRNA levels of the mentioned genes were measured by qPCR from total RNA extracted from wild-type and *Flp1-9A-GFP* strains growing in YES medium containing 1mM H_2O_2. Total RNAs were prepared

from treated cells at the time points indicated. mRNA levels were normalized with ribosomal 18S mRNA and determined with respect to time 0′ value, which was considered as 1. Data represent the average of two biological replicates.

Supplementary Figure S5. Δ*flp1* cells show greater resistance to moderate oxidative stress conditions. Asynchronous cultures of indicated strains growing at 30ºC in YES were incubated with 0.2 mM of H_2O_2 during 1 h. Then, cells were stressed with 2 mM (a) or 25 mM (b) H_2O_2. Cell viability was measured at the time points indicated by plating the appropriate dilution of cells onto YES agar plates. The number of viable cells was measured after 3 days incubation at 30ºC. Viability is expressed as a percentage of the number of colonies obtained before the addition of the first dose of H_2O_2.

Supplementary Figure S6. Protein levels of Pyp2 phosphatase upon oxidative stress induction. Wild-type and Δ*flp1*cells expressing a Pyp2-Myc fusion protein were grown in YES medium to mid-log phase and treated with 1mM H_2O_2 for the indicated times. Total extracts were resolved by SDS-PAGE and analyzed by Western blot. Pyp2 was detected by incubation with anti-Myc antibody. Tubulin was used as loading control. Normalization of quantified Pyp2 is shown in bar diagrams. Experiment was repeated three times (Supplementary information Fig. S11), and a representative experiment is shown. Blot membranes were cut prior antibodies incubation.

Supplementary Figure S7. Viability of Δ*pcr1 and* Δ*flp1* Δ*pcr1* mutants in response to oxidative stress. (a) Tenfold serial dilution of indicated strains were spotted onto YES-agar plates without or with 0.6 mM and 1mM H_2O_2 and incubated at 30ºC during 72 h. (b) Asynchronous cultures of indicated strains growing at 30ºC in YES were incubated with 2 mM of H_2O_2 during 1 h. Cell viability was measured before and after treatment by plating the appropriate dilution of cells onto YES agar plates. The number of viable cells was measured after 3 days incubation at 30ºC. Viability is expressed as a percentage of the number of colonies obtained before the addition of H_2O_2 (0′). Data represent the average of two biological replicates.

Supplementary Figure S8. Full-length gels and blots relating to Figure 2b,c are shown.
Supplementary Figure S9. Full-length gel and blot relating to Figure S3b are shown.
Supplementary Figure S10. Unprocessed original scans and full-length immunoblots relating to Figure 5a,b are shown.

Supplementary Figure S11. Unprocessed original scans and full-length blots relating to Figure S6 are shown.

Supplementary Figure S12. Full-length immunoblots relating to Figure 6a are shown.

Supplementary Figure S13. Unprocessed original scans and full-length immunoblots relating to Figure 7 are shown.

Supplementary Table S1

**Genotype**	**Strain**	**Source**
h^{+}*ade6-704 leu1-32 ura4-d18*	47.38	Lab stock
h^{+}*flp1::KanMX6 ade6-704 leu1-32 ura4-d18*	43.56	Lab stock
h^{-} *wis1::ura4*$^{+}$ *leu1-32 ura4-d18*	43.69	Dr. Shiozaki
h^{-} *flp1::KanMX6 wis1::ura4*$^{+}$ *ade6-704 leu1-32 ura4-d18*	44.01	Lab stock
h^{-} *sty1::ura4*$^{+}$ *leu1-32*	49.57	Dr. Moreno
h^{-} *flp1::KanMX6 sty1::ura4*$^{+}$ *ade6-704 leu1-32 ura4-d18*	51.30	Lab stock
h^{-}*srk1::KanMX6 ade6-704 leu1-32 ura4-d18*	43.05	Dr. Aligué
h^{-} *flp1::KanMX6 srk1::KanMX6 ade6-704 leu1-32 ura4-d18*	43.13	Lab stock
h^{90} *pcr1::pcr1-3Flag-KanMX6 leu1-32 ade6-216 his2 ura4-d18*	47.47	Dr. Grewal
h^{+}*pcr1::pcr1-3Flag-KanMX6 flp1::KanMX6 leu1-32 ade6-216 his2 ura4-d18*	49.20	Lab stock
h^{+}*pcr1::pcr1-3Flag-KanMX6 flp1::flp1-HA-KanMX6 leu1-32 ade6-216 his2 ura4-d18*	45.42	Lab stock
flp1-GFP:kanR gar2-RFP:kanR ade6-M21X ura4-d18 leu1-32	52.10	Lab stock
clp1::6A-GFP:kanR gar2-mCherry:kanR ade6-M21X ura4-d18 leu1-32	90.5	Dr. Gould
flp1::9A-EGFP:kanR gar2-RFP:kanR ade6-M21X ura4-d18 leu1-32	52.15	Lab stock
flp1::9A-EGFP-leu2-KanMX6	50.69	Lab stock
h^{-} *flp1::flp1-9A-EGFP:KanMX6 pcr1::pcr1-3Flag-Hph leu1-32*	90.06	This study
flp1::KanMX, atf1::ura4, leu1-32, ura4D18, leu1-32, Sty1::Hph	90.13	This study
h^{-}*flp1::ura4, pcr1::KanMX6, leu1-32, ura4-d18, ade6-216, Sty1::Hph*	90.10	This study
pap1::KanMX6 flp1::KanMX6, leu1-32, ura4-d18, ade-, Sty1::Hph	90.11	This study
flp1::KanMX6 pyp2::pyp2-Myc:ura4	43.43	Lab stock
h^{-}*pcr1::KanMX6, leu1-32, ura4-d18, ade6-216*	52.64	Lab stock
h^{-}*pcr1::KanMX6 flp1::ura4 leu1-32, ura4-d18, ade6-216*	53.56	Lab stock

Supplementary Table S2

Gene	Primers
atf1^{+}	F: TCACCTGGTACTGCCAATTTAT<br>R: CCATTTACAACAGGCGGTTTAC
ctt1^{+}	F: CTCCTGTACGTGAACGCCAAT<br>R: GAATACGGCGACCAAGCTCTG
gpd1^{+}	F: GCTCACGAGAATGTCAAATAC<br>R: GTCTAGCGACCTCACGAACAT
hsp9^{+}	F: ATGTCTGATCCCGCAAGAAAG<br>R: CTTGGAGGAGTCAGGAGTCAT
pcr1^{+}	F: CTCACAATGCTCTCCACTCTC<br>R: TTGAGTAGGGCTAACGGAAAC
pyp2^{+}	F: CTACGATCGGTGCCTTCTTATC<br>R: GATTGACGACGTTGCTGGATT
srx1^{+}	F: CATTGTTGAGCTTGATATGTC<br>R: GCTGGAGGAGTTTTCCCTGTC
apt1^{+}	F: CTCGTTACTCGTTTGGGTGG<br>R: GCCATAAGACGCTTACGTCC
trr1^{+}	F: CTGCAGCCATCTATCTCGCC<br>R: GACCACCAGCAGCAATACCG
18S rRNA^{+}	F: TGTACTGTGAAACTGCGAATGGCTC<br>R: GCAAGGCCATGCGATTCGAA

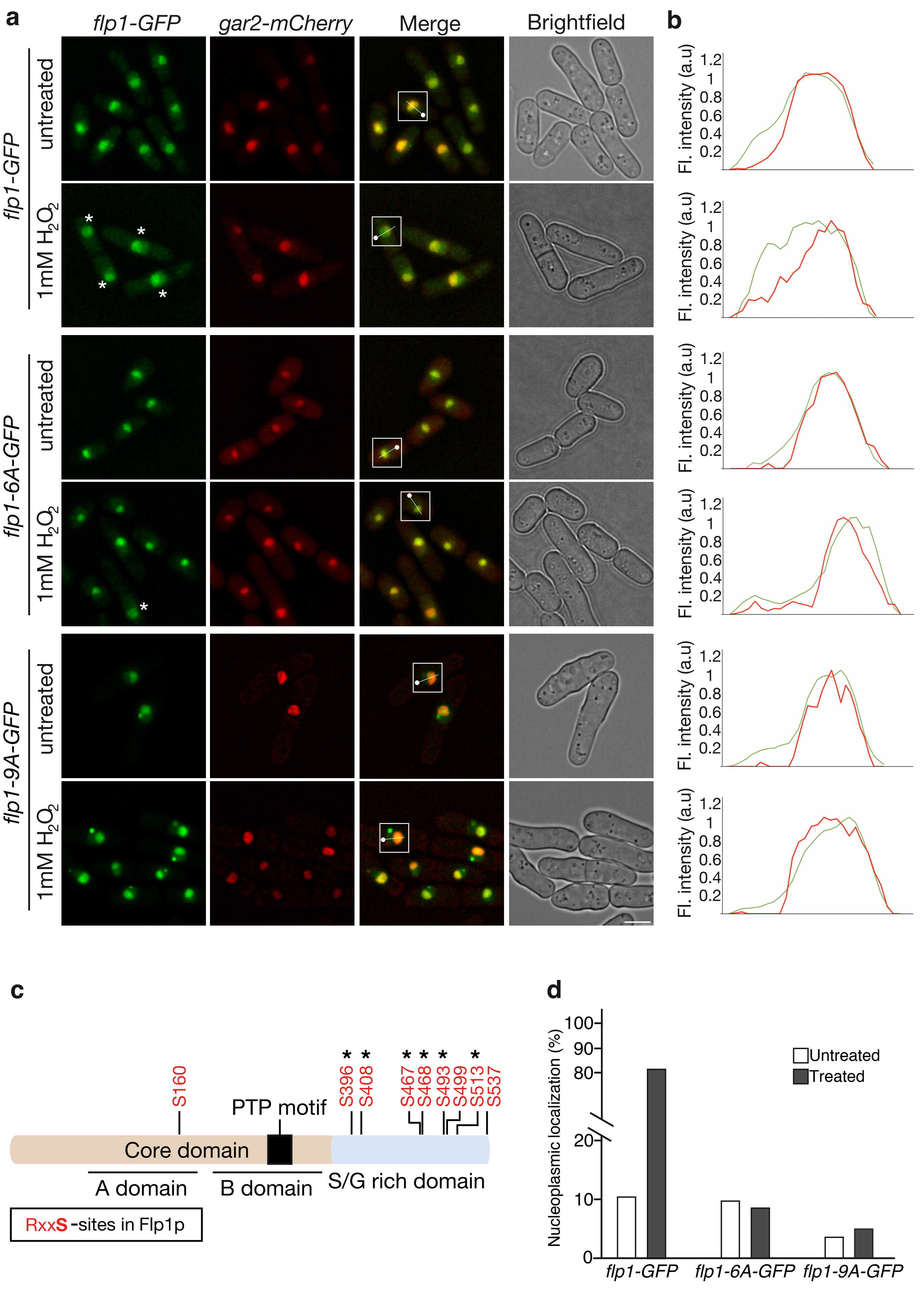
a
flp1-GFP
gar2-mCherry
Merge
Brightfield
b
flp1-GFP
untreated
1mM H_2O_2
flp1-6A-GFP
untreated
1mM H_2O_2
flp1-9A-GFP
untreated
1mM H_2O_2
Fl. intensity (a.u)
1.2
1
0.8
0.6
0.4
0.2
c
S160
PTP motif
S396
S408
S467
S468
S493
S499
S513
S537
Core domain
A domain
B domain
S/G rich domain
RxxS -sites in Flp1p
d
Nucleoplasmic localization (%)
100
90
80
20
10
0
Untreated
Treated
flp1-GFP
flp1-6A-GFP
flp1-9A-GFP

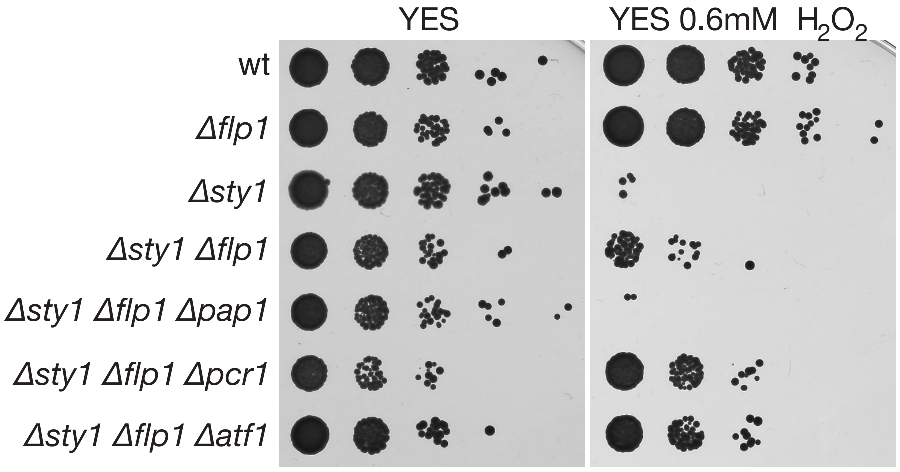
YES
YES 0.6mM H_2O_2
wt
Δflp1
Δsty1
Δsty1 Δflp1
Δsty1 Δflp1 Δpap1
Δsty1 Δflp1 Δpcr1
Δsty1 Δflp1 Δatf1

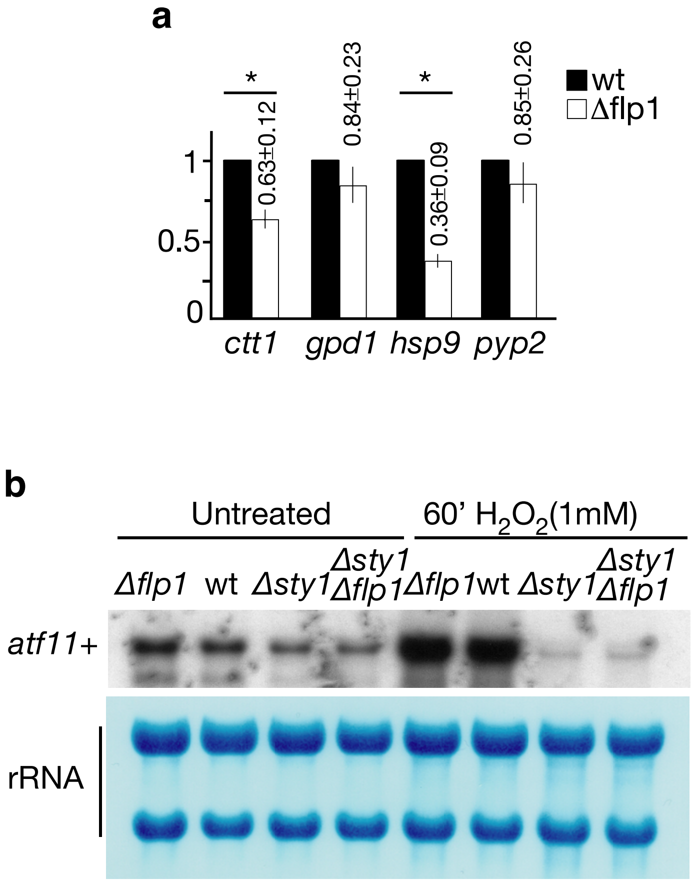
a
*
*
0.63±0.12
0.84±0.23
0.36±0.09
0.85±0.26
wt
Δflp1
1
0.5
0
ctt1
gpd1
hsp9
pyp2
b
Untreated
60' H_2O_2(1mM)
Δflp1
wt
Δsty1
Δsty1 Δflp1
Δflp1
wt
Δsty1
Δsty1 Δflp1
atf11+
rRNA

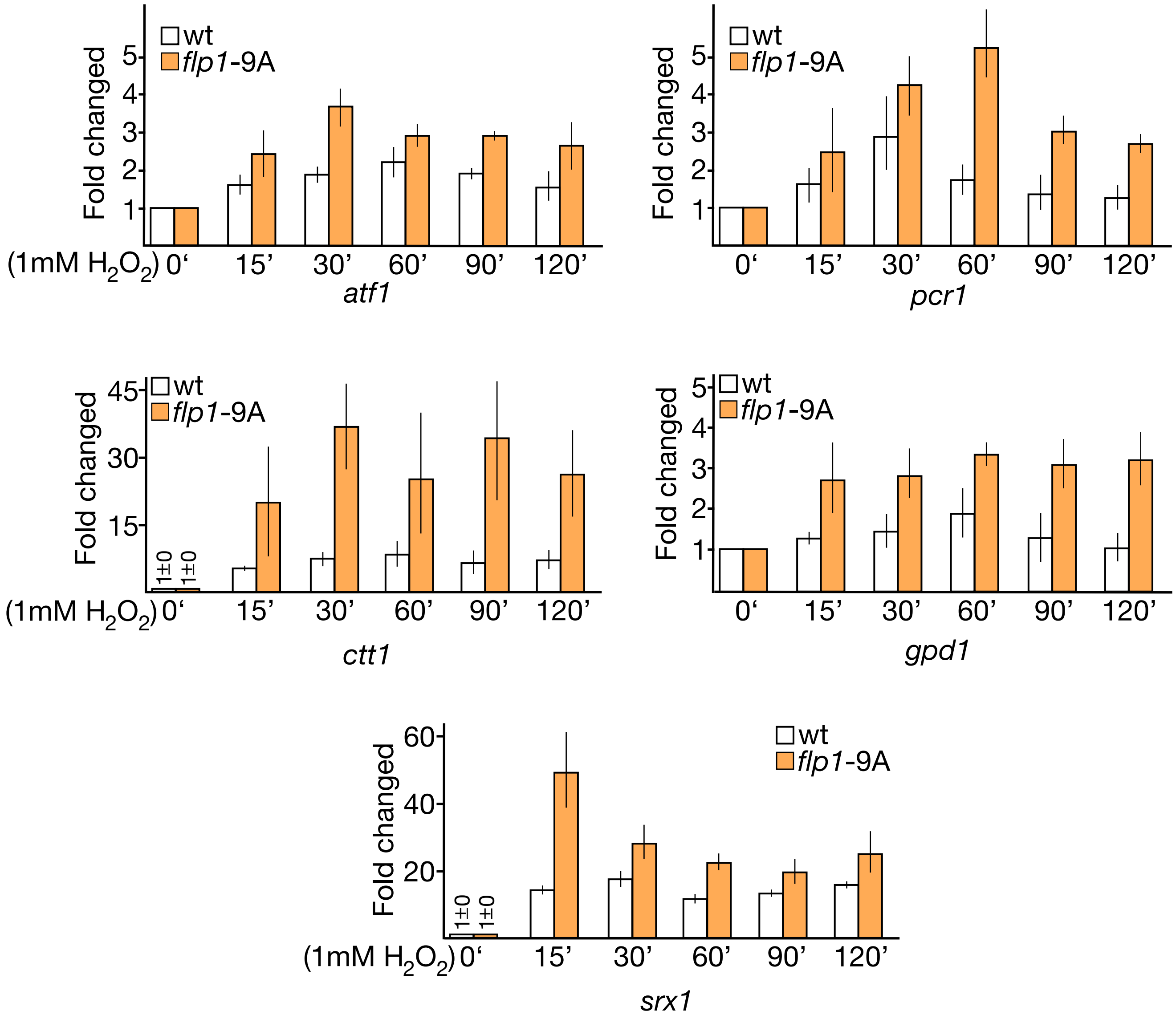

wt
flp1-9A
Fold changed
(1mM H2O2) 0' 15' 30' 60' 90' 120'
atf1
pcr1
1±0
ctt1
gpd1
srx1

a

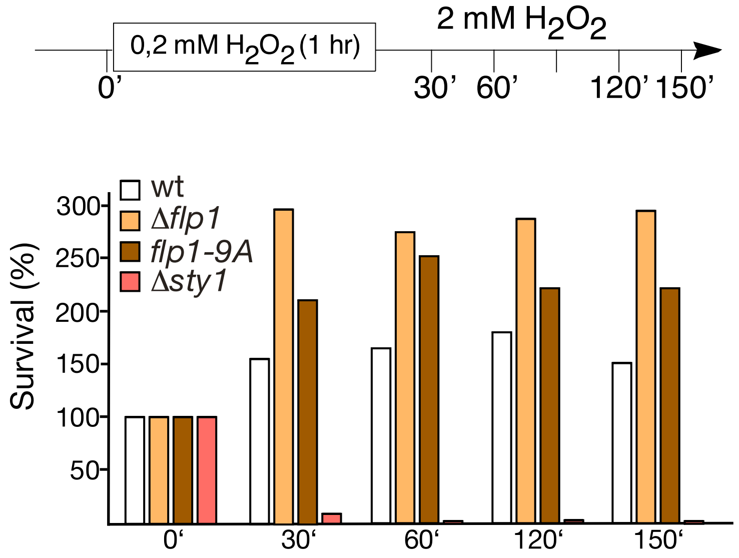
0,2 mM H_2O_2 (1 hr)
2 mM H_2O_2
0'
30'
60'
120'
150'
wt
Δ*flp1*
flp1-9A
Δ*sty1*
Survival (%)
300
250
200
150
100
50
0'
30'
60'
120'
150'

b

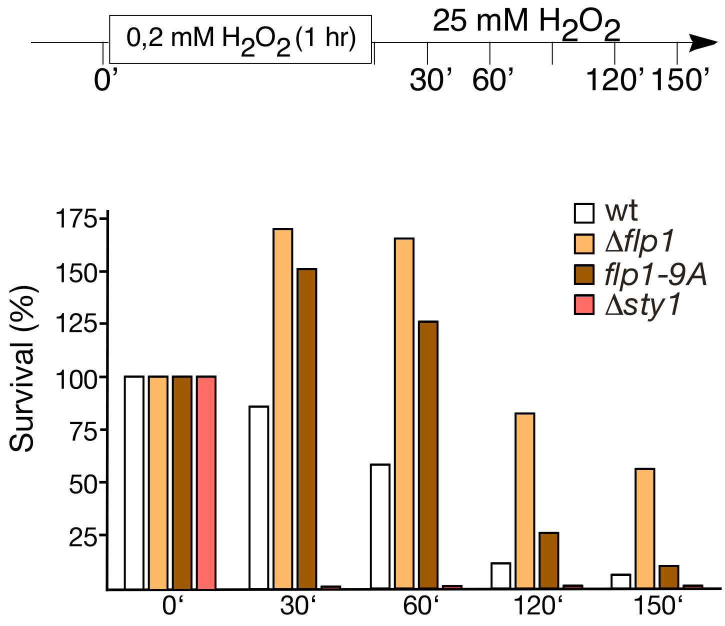
0,2 mM H_2O_2 (1 hr)
25 mM H_2O_2
0'
30'
60'
120'
150'
wt
Δ*flp1*
flp1-9A
Δ*sty1*
Survival (%)
175
150
125
100
75
50
25
0'
30'
60'
120'
150'

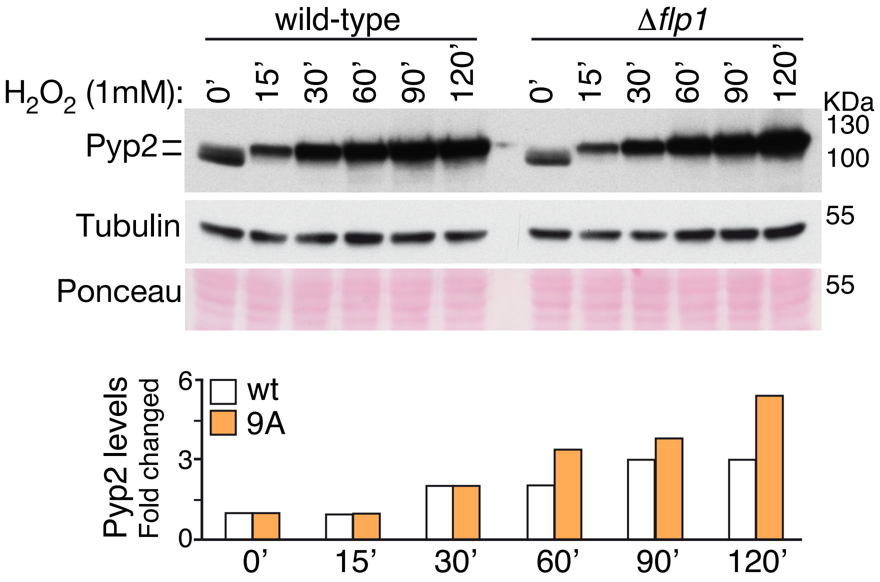
wild-type
Δflp1
H2O2 (1mM):
0’ 15’ 30’ 60’ 90’ 120’
0’ 15’ 30’ 60’ 90’ 120’
KDa
130
100
Pyp2
Tubulin
55
Ponceau
55
Pyp2 levels
Fold changed
6
3
0
wt
9A
0’
15’
30’
60’
90’
120’

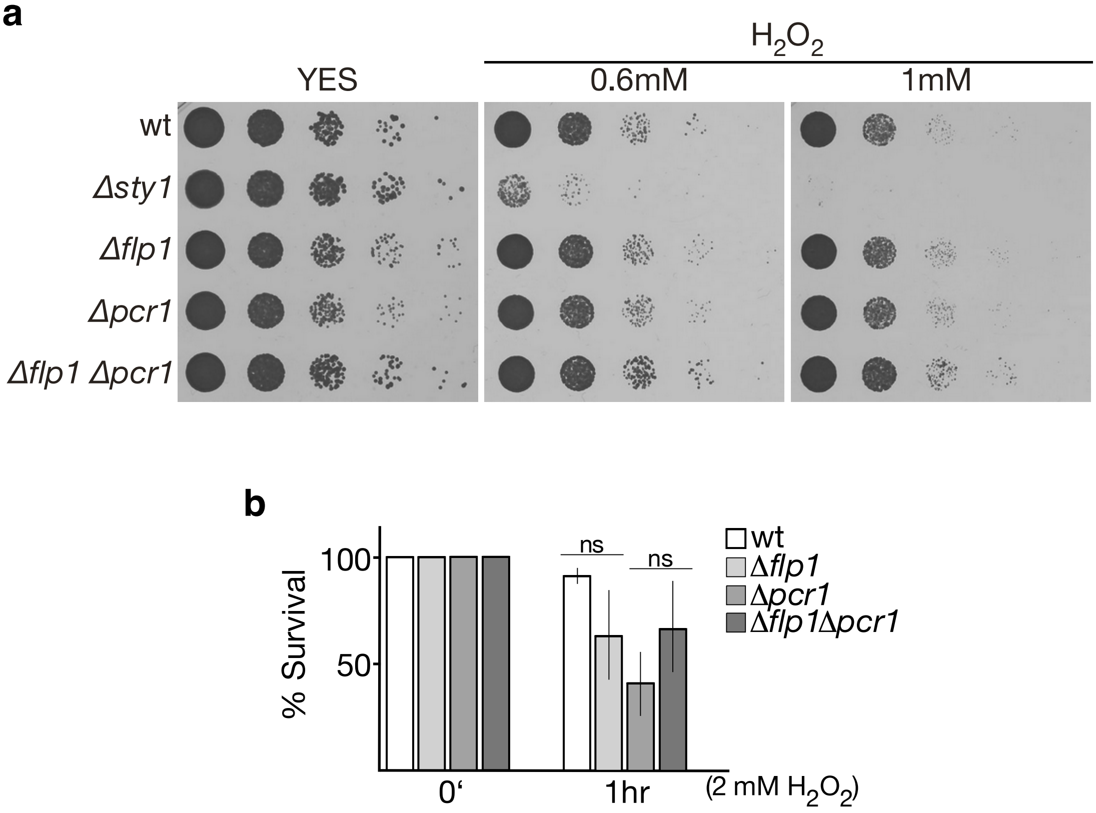
a
H_2O_2
YES
0.6mM
1mM
wt
Δsty1
Δflp1
Δpcr1
Δflp1 Δpcr1
b
% Survival
100
50
0'
1hr
(2 mM H_2O_2)
ns
ns
wt
Δflp1
Δpcr1
Δflp1Δpcr1

2b

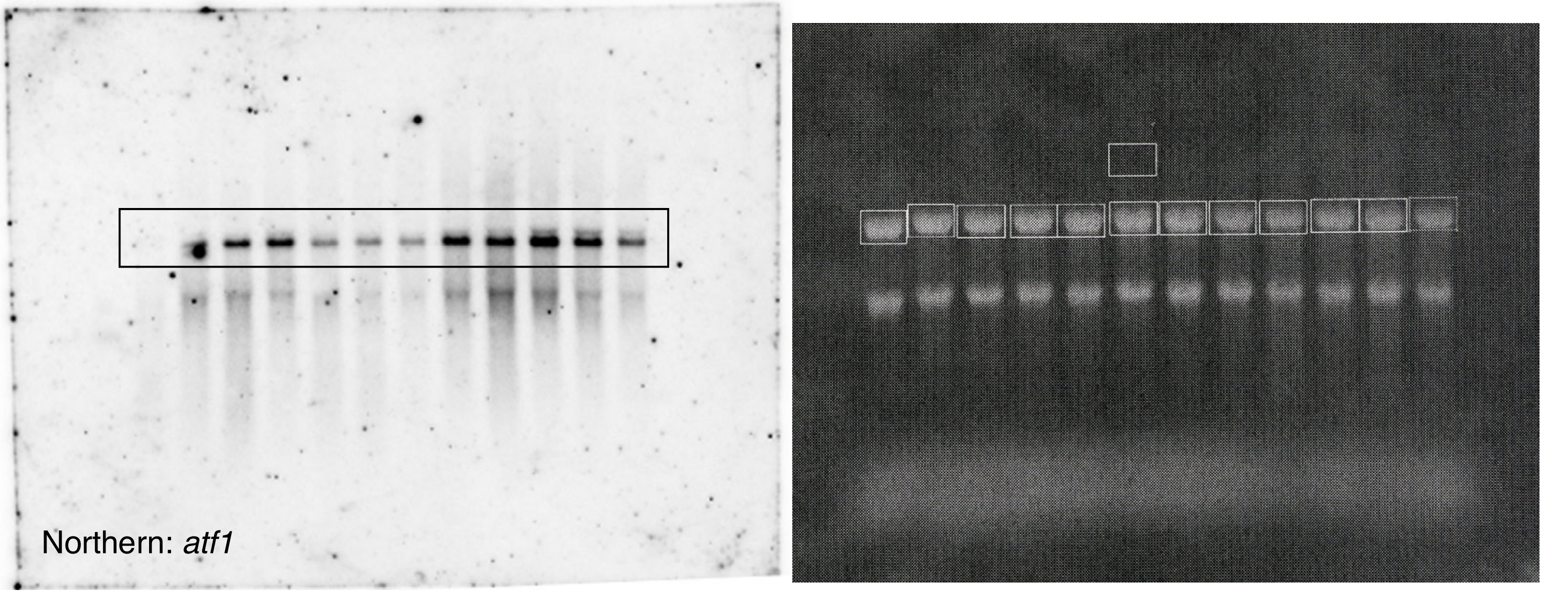

rRNA gel

2c

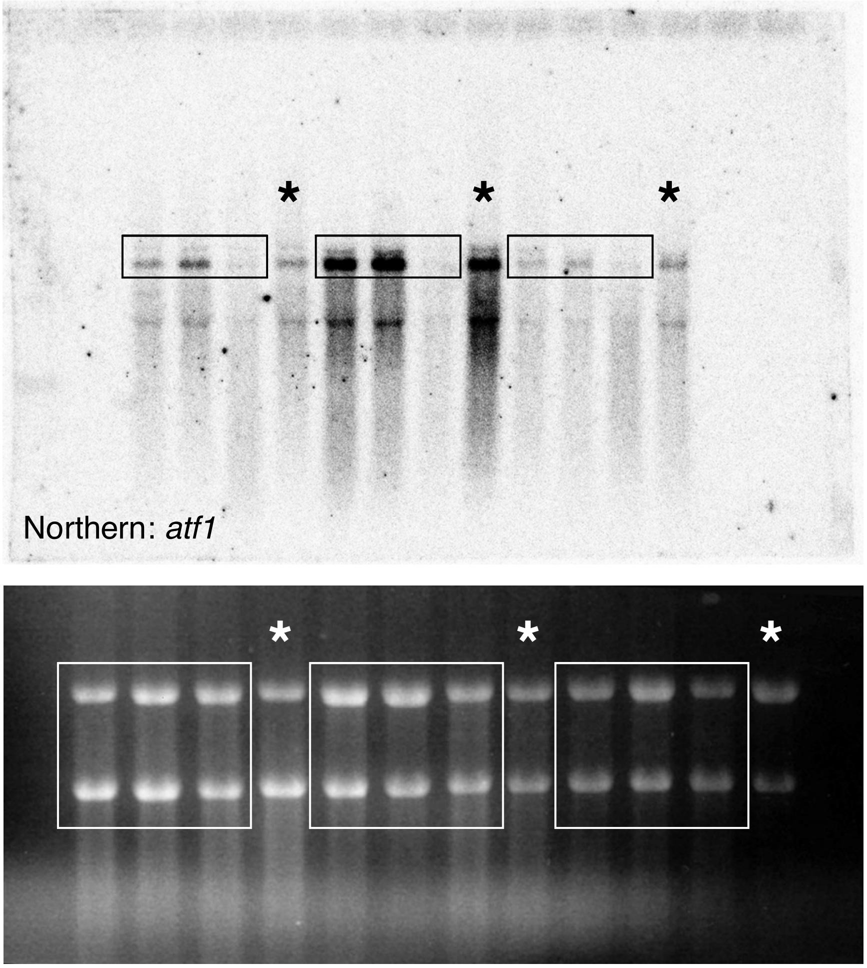

rRNA gel

*unrelated samples

S3b

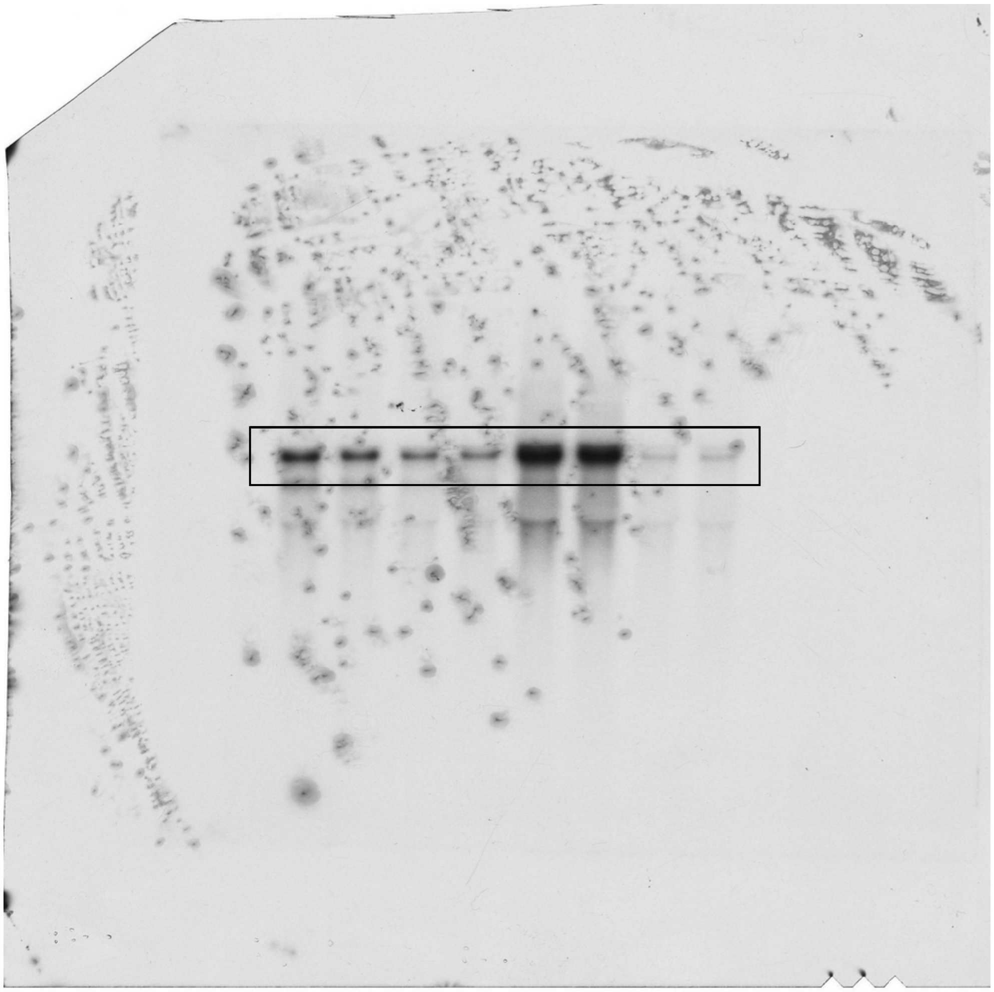

Northern: *atf1*

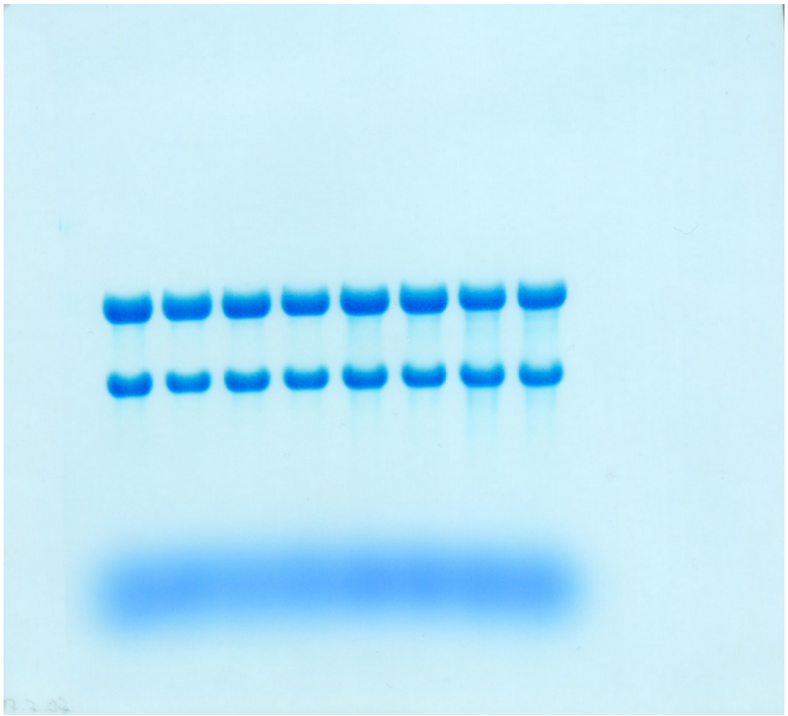

rRNA staining

5a

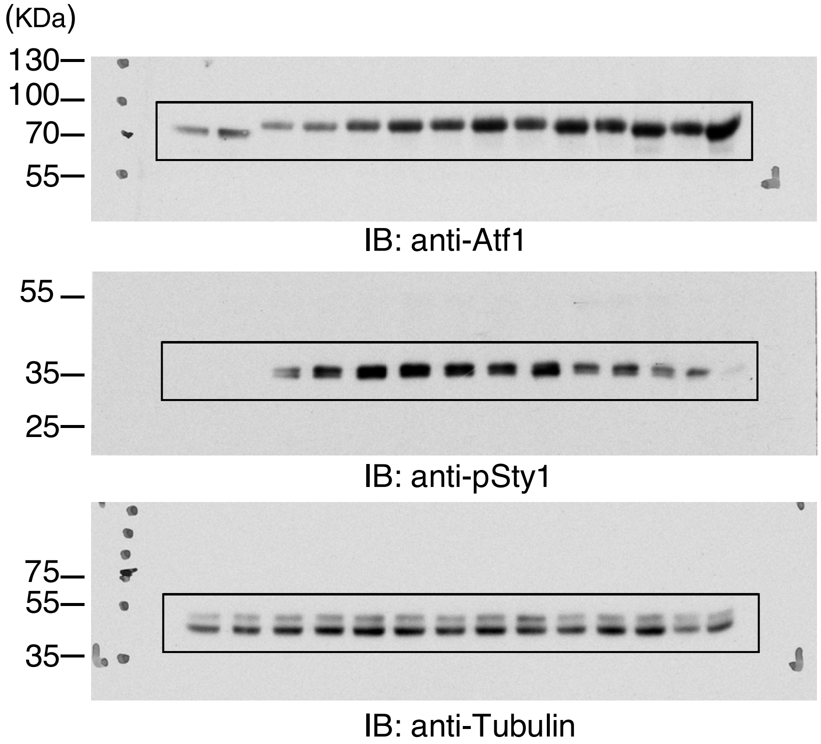

5b

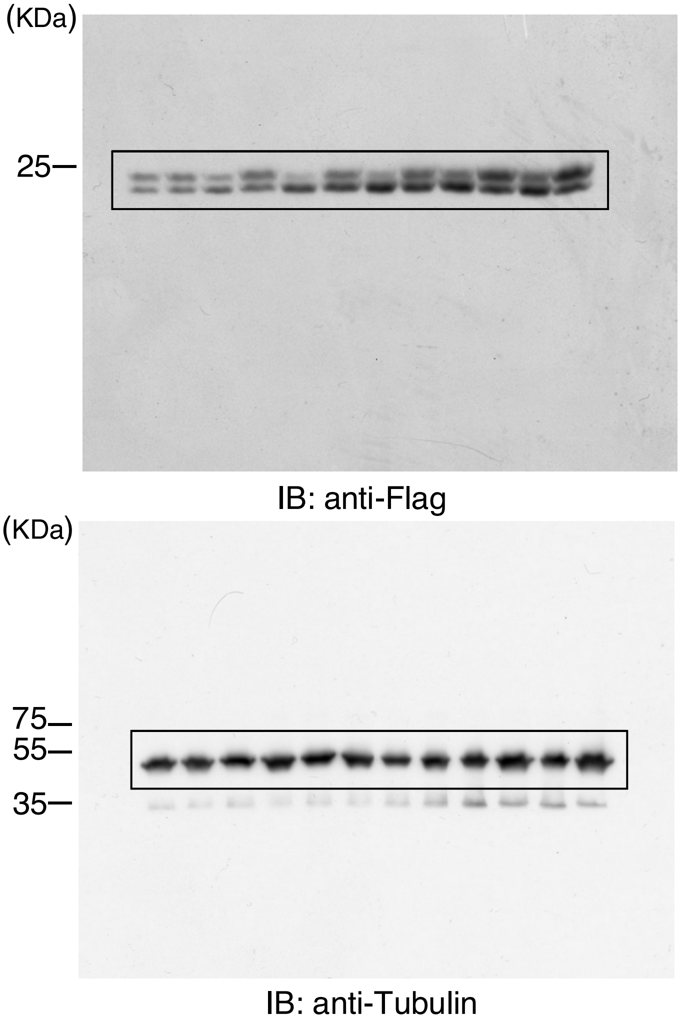

IB: Immunoblot

S6

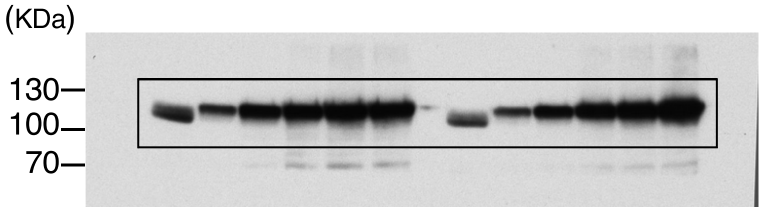

IB: anti-Pyp2

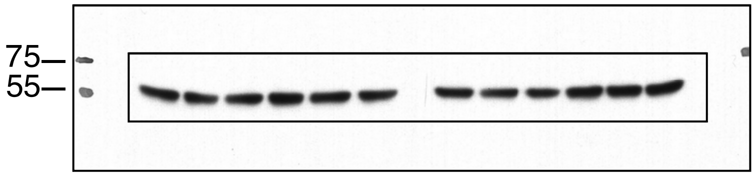

IB: anti-Tubulin

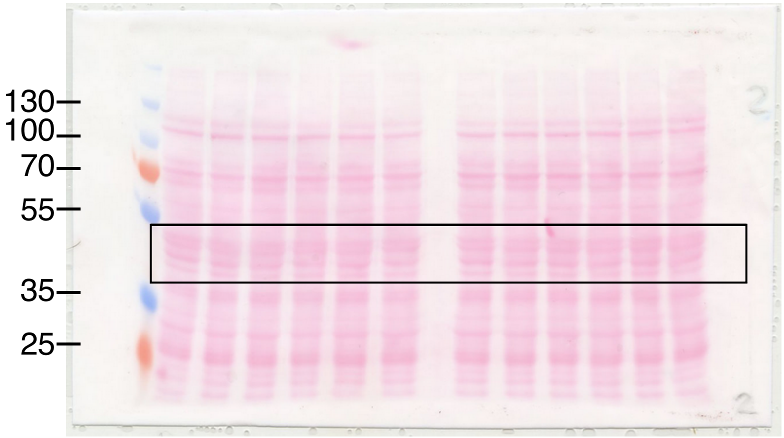

Ponceau staining

Replicate 1

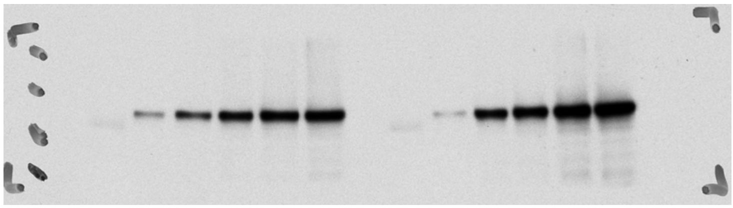

IB: anti-Pyp2

Replicate 2

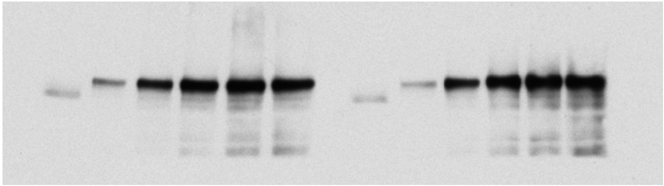

IB: anti-Pyp2

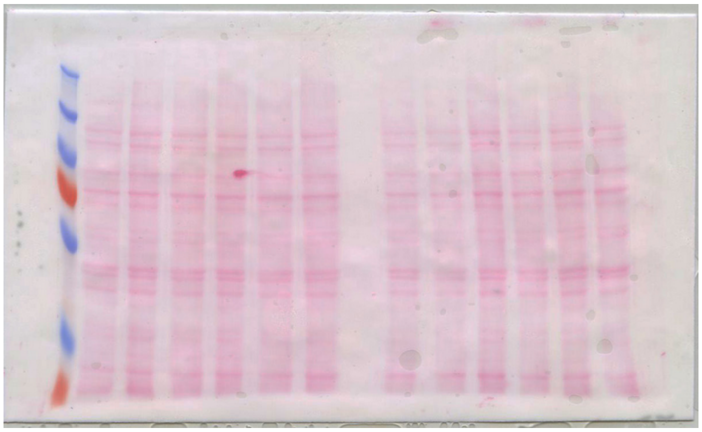

Ponceau staining

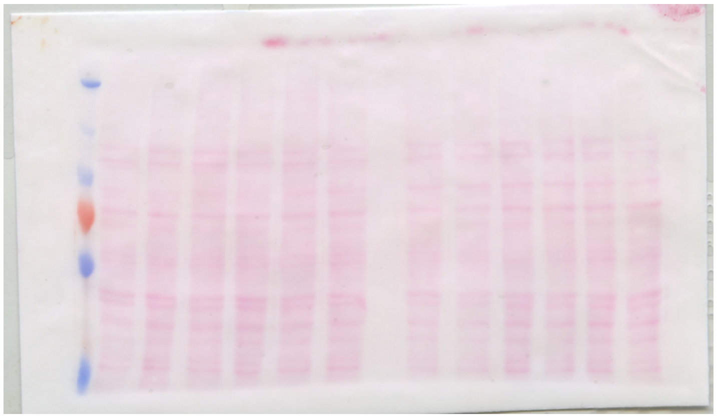

Ponceau staining

IB: Immunoblot

6a

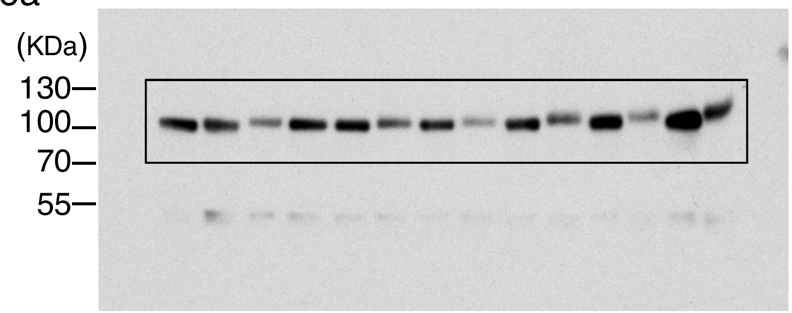

IB: anti-Atf1

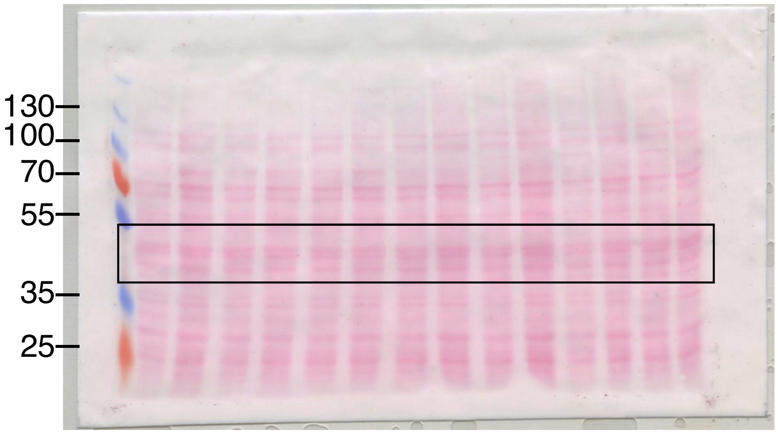

Ponceau staining

IB: Immunoblot

7a

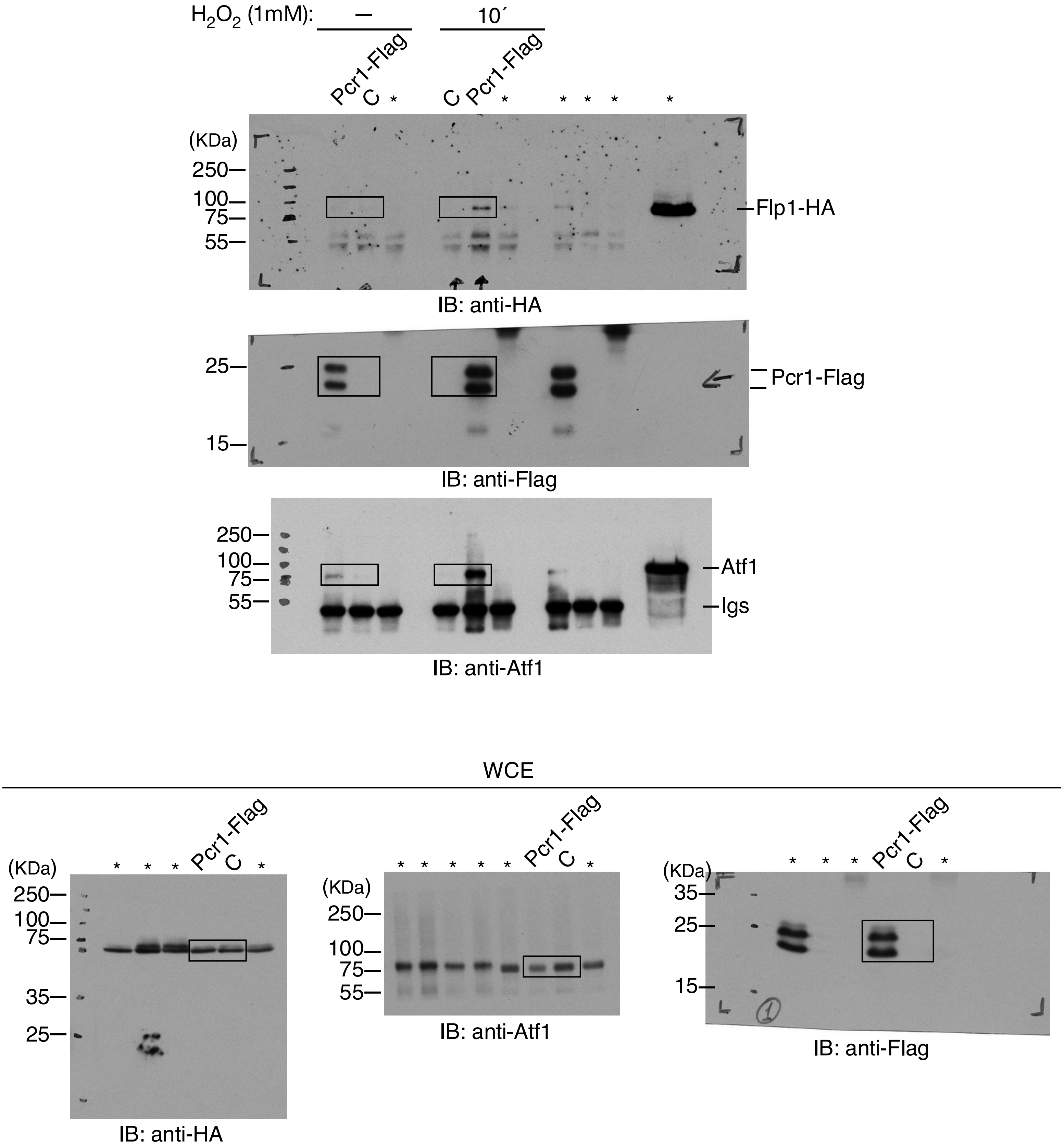

IB, Immunoblot

(*) samples not shown (unrelated samples)

7b

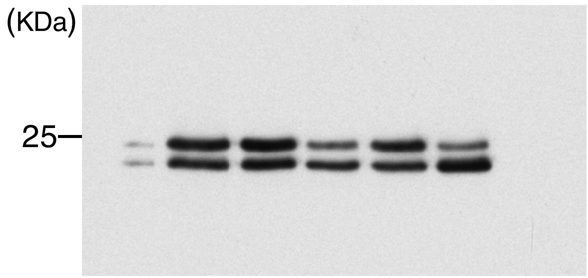

IB: anti-Pcr1

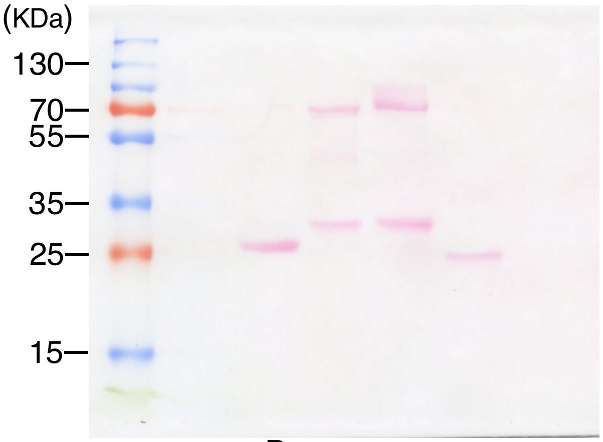

Ponceau

IB, Immunoblot
